# Transcriptome analysis of *WRKY* gene family in *Oryza officinalis* Wall ex Watt and *WRKY* genes involved in responses to *Xanthomonas oryzae* pv. *oryzae* stress

**DOI:** 10.1371/journal.pone.0188742

**Published:** 2017-11-30

**Authors:** Chunmiao Jiang, Qingxi J. Shen, Bo Wang, Bin He, Suqin Xiao, Ling Chen, Tengqiong Yu, Xue Ke, Qiaofang Zhong, Jian Fu, Yue Chen, Lingxian Wang, Fuyou Yin, Dunyu Zhang, Walid Ghidan, Xingqi Huang, Zaiquan Cheng

**Affiliations:** 1 Biotechnology & Genetic Germplasm Institute, Yunnan Academy of Agricultural Sciences, Kunming, Yunnan, P.R. China; 2 School of Life Sciences, Yunnan University, Kunming, Yunnan, P.R. China; 3 School of Life Sciences, University of Nevada, Las Vegas, USA; 4 Jiangxi Science & Technology Normal University, Nanchang, Jiangxi, P.R. China; 5 Rice Research & Training Center, Field Crops Research Institute, Agricultural Research Center (ARC), Sakha, Kafr Elsheikh, Egypt; ICAR-Indian Institute of Rice Research, INDIA

## Abstract

*Oryza officinalis* Wall ex Watt, a very important and special wild rice species, shows abundant genetic diversity and disease resistance features, especially high resistance to bacterial blight. The molecular mechanisms of bacterial blight resistance in *O*. *officinalis* have not yet been elucidated. The *WRKY* transcription factor family is one of the largest gene families involved in plant growth, development and stress response. However, little is known about the numbers, structure, molecular phylogenetics, and expression of the *WRKY* genes under *Xanthomonas oryzae* pv. *oryzae* (*Xoo*) stress in *O*. *officinalis* due to lacking of *O*. *officinalis* genome. Therefore, based on the RNA-sequencing data of *O*. *officinalis*, we performed a comprehensive study of *WRKY* genes in *O*. *officinalis* and identified 89 *OoWRKY* genes. Then 89 *OoWRKY* genes were classified into three groups based on the WRKY domains and zinc finger motifs. Phylogenetic analysis strongly supported that the evolution of *OoWRKY* genes were consistent with previous studies of *WRKYs*, and subgroup IIc *OoWRKY* genes were the original ancestors of some group II and group III *OoWRKYs*. Among the 89 *OoWRKY* genes, eight *OoWRKY*s displayed significantly different expression (>2-fold, p<0.01) in the *O*. *officinalis* transcriptome under *Xoo* strains PXO99 and C5 stress 48 h, suggesting these genes might play important role in PXO99 and C5 stress responses in *O*. *officinalis*. QRT-PCR analysis and confirmation of eight *OoWRKYs* expression patterns revealed that they responded strongly to PXO99 and C5 stress 24 h, 48 h, and 72 h, and the trends of these genes displaying marked changes were consistent with the 48 h RNA-sequencing data, demonstrated these genes played important roles in response to biotic stress and might even involved in the bacterial blight resistance. Tissue expression profiles of eight *OoWRKY* genes revealed that they were highly expressed in root, stem, leaf, and flower, especially in leaf (except *OoWRKY71*), suggesting these genes might be also important for plant growth and organ development. In this study, we analyzed the WRKY family of transcription factors in *O*.*officinalis*. Insight was gained into the classification, evolution, and function of the *OoWRKY* genes, revealing the putative roles of eight significantly different expression *OoWRKYs* in *Xoo* strains PXO99 and C5 stress responses in *O*.*officinalis*. This study provided a better understanding of the evolution and functions of *O*. *officinalis WRKY* genes, and suggested that manipulating eight significantly different expression *OoWRKYs* would enhance resistance to bacterial blight.

## Introduction

Since the first *WRKY* gene of sweet potato is reported in 1994 [1], many *WRKY* genes have been identified from a wide variety of plant genome [2–7]. A large majority of *WRKY* genes functions as a positive or negative regulator's response to various biotic and abiotic stresses in plant [8–9]. *WRKY* have been demonstrated to mediate the regulatory network of salicylic acid (SA), jasmonic acid (JA), gibberellins (GA), abscisic acid (ABA) and ethylene (ET) [10–15], and reported to play an important role in resistance against diseases caused by bacteria or fungi [16]. In rice, 35 *WRKYs* of which *OsWRKY3*, *OsWRKY13*, *OsWRKY28*, *OsWRKY30*, *OsWRKY31*, *OsWRKY45*, *OsWRKY53*, *OsWRKY70* and *OsWRKY71* play important regulatory role in disease resistance-related pathways of bacterial blight, rice blast and sheath blight, and are induced by pathogen [17]. Over-expressing *OsWRKY53* and *OsWRKY71* enhanced the resistance of transgenic rice against fungal blast and bacterial blight, respectively [18–19]. In addition, *WRKY* genes also play an important role in the control of leaf senescence, seed formation, dormancy and germination [11, 20–21].

*WRKY* transcription factors, which have a significant character, contain one or two WRKY domains that are composed of a WRKY motif and a zinc finger motif [3]. The core sequence of a WRKY motif is WRKYGQK with some variants including WRKYGKK, WRKYGEK, WRKYGRK, WKKYGQK, WKRYGQK and WSKYEQK. Zinc fingers include two types of C_2_H_2_ motif (C-X_4-5_-C-X_22-23_-H-X_1_-H) and C_2_HC motif (C-X_5–7_-C-X_23_-H-X_1_-C) [3]. Based on the number of WRKY domains and zinc finger motifs, *WRKY* genes are usually divided into Ⅰ, Ⅱ and Ⅲ group [2]. Group Ⅰ has two WRKY domains, and is divided into two subgroups, Ia containing C_2_H_2_ zinc fingers and Ib containing C_2_HC zinc fingers; group Ⅱ, which containing one WRKY domain with C_2_H_2_ zinc finger, are divided into subgroup Ⅱa, Ⅱb, Ⅱc, Ⅱd and Ⅱe based on their phylogenetic relationship; group III has one WRKY domain with C_2_HC zinc finger. After analysis of *WRKY* transcription factor family in *Arabidopsis thaliana* and rice genomes, the classification of *WRKY* genes group II is further optimized that the subgroup Ⅱa, Ⅱb, Ⅱd and Ⅱe merged into Ⅱa+b and Ⅱd+e [3–4].

*Oryza officinalis* Wall ex Watt is one of the important and special wild rice species in *Oryza* genus. It is one of the three wild species indigenous in China and shows abundant genetic diversity and disease resistance features, especially high resistance to bacterial blight [22–23]. The molecular mechanisms of bacterial blight resistance in *O*. *officinalis* have not yet been elucidated. *WRKY* genes play an important role in resistance against diseases caused by bacteria or fungi [16], but there is no analysis of *O*.*officinalis WRKY*s due to lack of genome information. Therefore, based on the *O*. *officinalis* transcriptome under the strains PXO99 and C5 of *Xanthomonas oryzae* pv. *oryzae* (*Xoo*) stress, 89 genes encoding WRKY transcription factors (*OoWRKY*) were identified, and the analysis of their structure, molecular phylogenetics, conserved motifs, and stimulation in response to *Xoo* strains PXO99 and C5 were performed. The results provided insights into the evolution of *O*. *officinalis WRKYs* and their functions in *Xoo* stress responses. To our knowledge, this is the first report of *O*. *officinalis WRKY* genes.

## Materials and methods

### Plant materials and treatments

*Oryza officinalis* Wall ex Watt (CC, 2n = 2x = 24), strains PXO99 and C5 of *Xanthomonas oryza* pv. *oryza* (*Xoo*) were provided by the Biotechnology & Genetic Germplasm Institute, Yunnan Academy of Agricultural Sciences, Yunnan, China. PXO99 was a Philippines hypervirulent *Xoo* strain, and C5 was the hypervirulent and respresentative strain in China. The *O*. *officinalis* had strong resistance to C5, but its resistance to PXO99 was weak (unpulished data). *Xoo* strains PXO99 and C5 were used to inoculate *O*. *officinalis*.

The PXO99 and C5 were cultured on Nutrient Agar medium at 28°C for 48 h. The bacterial strains were suspended in sterile water, adjusted the concentration of bacterial liquid to OD_600_ = 0.8~1.0 using the NanoDrop2000, and then used to inoculate *O*. *officinalis* leaves. *O*. *officinalis* were respectively inoculated with strains PXO99 and C5 by leaf-clipping method [24] at 14: 00~15: 00 under 28~30°C room temperature. The control experiment of plant leaves were cut with sterile water (ddH_2_O) instead of bacterial liquid by the same method. The treated leaves were collected at 48 h and used for RNA-seq. In addition, the treated leaves were collected at 0 h, 24 h, 48 h, 72 h, 96 h, 120 h respectively, and used for analysis of *O*. *officinalis WRKY* genes expression in response to *Xoo* strains PXO99 and C5 by qRT-PCR.

For analysis of *WRKY* genes expression in different organs using qRT-PCR, the root, stem, leaf and flower of *O*. *officinalis* were collected under normal growth conditions and used for extraction of total RNA.

### Analysis of *O*. *officinalis WRKY* genes expression in response to *Xoo* using transcriptomic data

Total RNA was extracted from the leaves inoculated respectively with ddH_2_O, strains PXO99 and C5 for 48 h according to the manufacturer's instructions of RNeasy Plant Mini Kit and the RNase-Free DNase Set (Qiagen, Germany). RNA pools were constructed using 3 μg of RNA per sample according to the manufacturer’s instructions and sequenced on an Illumina HiSeq 4000 (Illumina, Inc., San Diego, CA, USA) next-generation platform technology. Gene expression levels were calculated in FPKM (Fragments Per Kilobase Per million Fragments mapped) and the FPKM values for each gene in all samples were log10 transformed [25]. Finally, a heat map was generated using TreeView software [26].

### Identification of the *O*. *officinalis WRKY* genes

BLAST analysis with the *O*. *sativa ssp*. *Japonica WRKY* genes were used to check the predicted *WRKY* genes from the *O*. *officinalis* database. A hidden Markov model (HMM) was constructed using the *O*. *sativa* WRKY amino acid sequences [27]. All the potential *O*. *officinalis* WRKY proteins were used to identify by HMMER3.1 and BLAST if they contained a WRKY domain. And then the CDD and PFAM databases were used to validate all the potential *O*.*officinalis* WRKY genes.

### Classification of the *O*. *officinalis WRKY* genes

The protein sequences of *O*. *officinalis WRKY* genes were aligned by Clustal X2.1 and classified into groups based on the numbers of WRKY domains and zinc finger motifs [2–4]. In brief, *O*. *officinalis WRKY* genes were divided into groups' Ⅰ, Ⅱ and Ⅲ. Group Ⅰ contained two WRKY domains and was divided into two subgroups based on zinc finger types, including subgroup Ⅰa with C_2_H_2_ zinc finger and subgroup Ⅰb with C_2_HC zinc finger. The group Ⅱ contained one WRKY domain with C_2_H_2_ zinc finger. Group Ⅲ contained one WRKY domain with C_2_HC zinc finger.

### The analysis of protein sequence motifs in *O*. *officinalsis WRKY* proteins

MEME (http://meme-suite.org/tools/meme) was used to predict and analyze motifs of *O*. *officinalis WRKY* proteins [28]. The numbers of motif were chosen 10 motifs; the motif widths were set between 6 and 50. The other parameters were set to default values.

### Phylogenetic analysis

The WRKY domain and full-length protein sequence of *O*. *officinalis WRKY* genes were used to analyze their phylogenetic relationship. The multiple protein sequences of *O*.*officinalis WRKY* genes were aligned by MUSCLE in MEGA6.0 with default parameters. The neighbor-joining (NJ) phylogenetic trees were constructed based on the aligned results of *O*. *officinalis* WRKY domain and full-length protein sequences using MEGA6.0 with bootstrap replications of 1000.

### QRT-PCR analysis of *O*. *officinalis WRKY* genes expression in response to *Xoo* and in different organs

The total RNA was extracted with the Omega plant RNA kit (Omega Bio-Tek, Georgia, USA) according to the instructions provided by the manufacturer. A total of 1 μg of RNA was reverse-transcribed into cDNA using PrimeScript RT reagent with the gDNA Eraser kit (TaKaRa, Dalian, China). A control amplicon was generated using the *β*-actin primers for amplification of *β*-actin (S1 Table). The primers of *O*. *officinalis WRKY* genes were designed by the online tool (https://www.ncbi.nlm.nih.gov/tools/primer-blast) (S1 Table). Gene expression levels were determined by perfoming quantitative real-time polymerase chain reaction (qRT-PCR) in Applied Biosystems QuantStudio 6 Flex (ABI, USA) using SYBR Premix Ex Taq II (TaKaRa) according to the manufacturer's instructions. Datas were analyzed by QuantStudio 6 Flex software (ABI, USA) and the 2^-△△CT^ method [29].

## Results

### Identification of *OoWRKY* genes in *O*. *officinalis*

All the *O*. *sativa ssp*. *Japonica* WRKY protein sequences were used as queries for the BLAST to identify *O*. *officinalis WRKY* proteins. HMM search was also performed against the *O*. *officinalis* proteins using WRKY domains. In total, 89 *WRKY* genes were identified after excluding the *WRKYs* of repeatable sequences and incomplete WRKY domains. These 89 *WRKY* genes were named *OoWRKY1* to *OoWRKY125* corresponding to the names of their orthologs in *O*. *sativa*. The details of these *OoWRKY* genes, such as their locus numbers, types of the encoded WRKY domains and sizes of the deduced peptides, were listed in S2 Table. The 89 *OoWRKY* proteins ranged from 141 (*OoWRKY60*) to 1,252 (*OoWRKY125*) amino acids (aa) in length, with an average length of approximately 370 aa. The MSU IDs of the *O*. *sativa* orthologs can also be found in S2 Table.

### Classification and phylogenetic analysis of the *OoWRKY* genes

**Fig 1:**
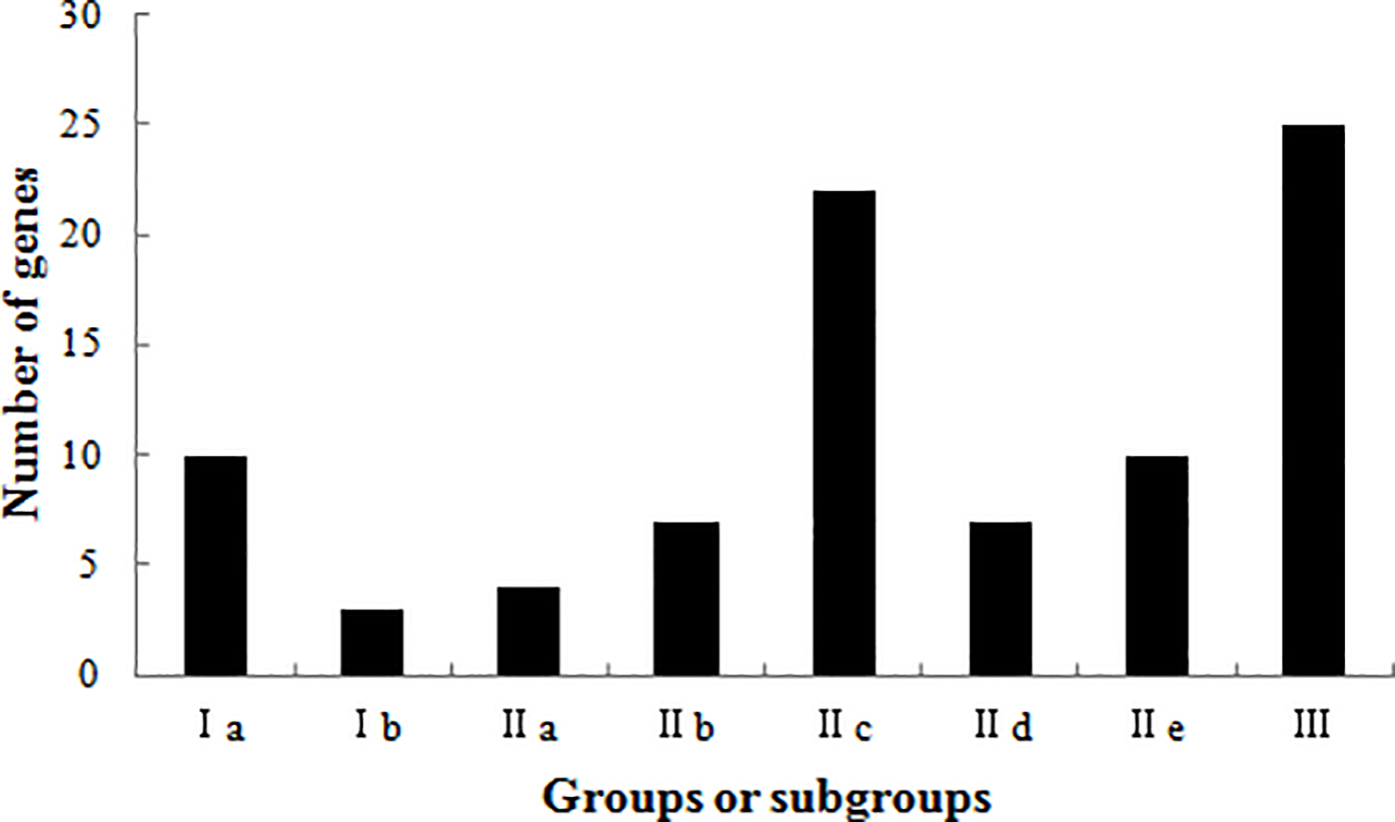
The distribution of the *OoWRKY* genes based on groups or subgroups.

The 89 *OoWRKY* genes were classified into different groups and subgroups according to published methods [2, 27]. Thirteen *OoWRKY* genes with two WRKY domains were belong to group I, which further divided into Ia and Ib based on the zinc finger motifs of C_2_H_2_-type (C-X_4–5_-C-X_22–23_-H-X_1_-H) and C_2_HC-type (C-X_7-10_-C-X_23_-H-X_1_-C), respectively; 50 group II *OoWRKY* genes, containing a C_2_H_2_-type (C-X_4–5_-C-X_22–24_-H-X_1_-H) zinc finger motif, were divided into five subgroups, including 11 group IIa+b, 22 group IIc and 17 group IId+e; 25 *OoWRKY* genes were belong to group III with a C_2_HC-type (C-X_5–7_-C-X_23–33_-H-X_1_-C) zinc finger motif (Fig 1). In addition, *OoWRKY118* was not able to classify into any other groups, although it had a WRKY domain and a zinc finger structure. All the WRKY domains and the zinc finger sequence of *OoWRKY* genes were showed as S1 File.

**Fig 2:**
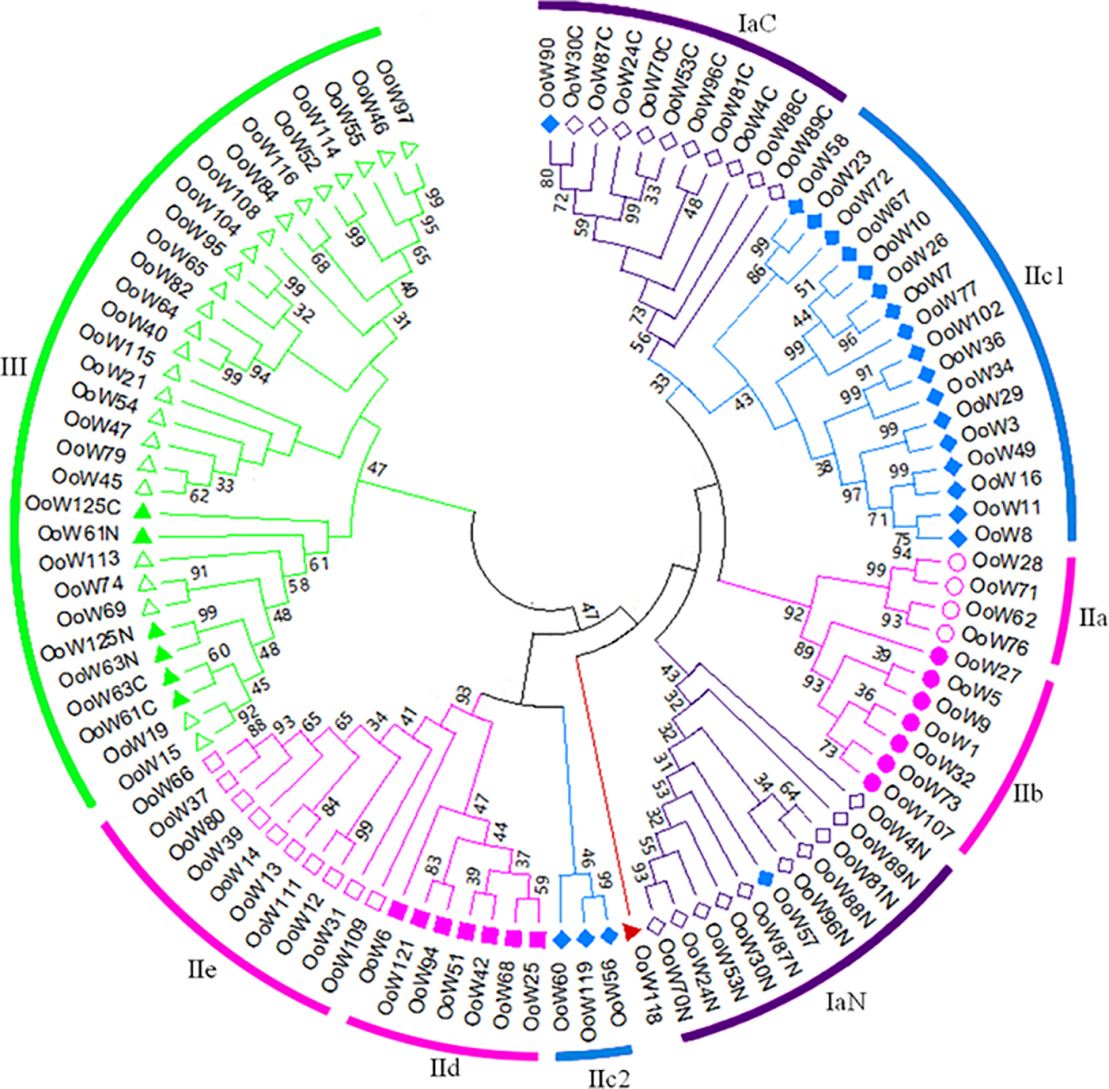
Phylogenetic analysis of the *OoWRKY* domains. The WRKY domain sequences were aligned by MUSCLE in the MEGA6 using the default parameters. The consensus NJ tree was shown by the results of 1,000 bootstrap replications. Bootstrap values were displayed with nodes. Group Ia: hollow diamond; Group Ib: filled triangle; Group IIa: circle; Group IIb: filled circle; Group IIc: filled diamond; Group IId: filled square; Group IIe: square; Group III: triangle; OoWRKY118: red filled triangle.

In order to analysis of the phylogenetic relationship of the *OoWRKYs*, a neighbor-joining (NJ) phylogenetic tree was constructed by using MEGA6.0 for the multiple sequence alignment of all *OoWRKY* domains with 1000 bootstrap analysis (Fig 2, S2 File). The NJ tree of *OoWRKY* domains was clustered into three groups (I, II, and III) (Fig 2). The N-terminal domains and C-terminal domains of subgroup Ia *OoWRKYs* were clustered into clade IaN and IaC respectively. Group II was diverged into four clades, of which subgroups IIa and IIb clustered into one clade, subgroups IId and IIe clustered into another, and subgroup IIc clustered into IIc1 and IIc2 clades. Most of the subgroup IIc *OoWRKY* domains were clustered into IIc1 neighbored the IaC clade, while the subgroup IIc *OoWRKY90* and *OoWRKY57* were clustered within the IaC and IaN clades respectively, which demonstrated a close evolutionary relationship between the subgroup IIc and subgroup Ia genes. The Group III *OoWRKY* genes were clustered into one clade including three subgroup Ib *OoWRKYs*. *OoWRKY118* was scattered amongst the different clades, fell outside of IIa+b and IIc1 clades, which suggested it didn't belong to these subgroups.

**Fig 3:**
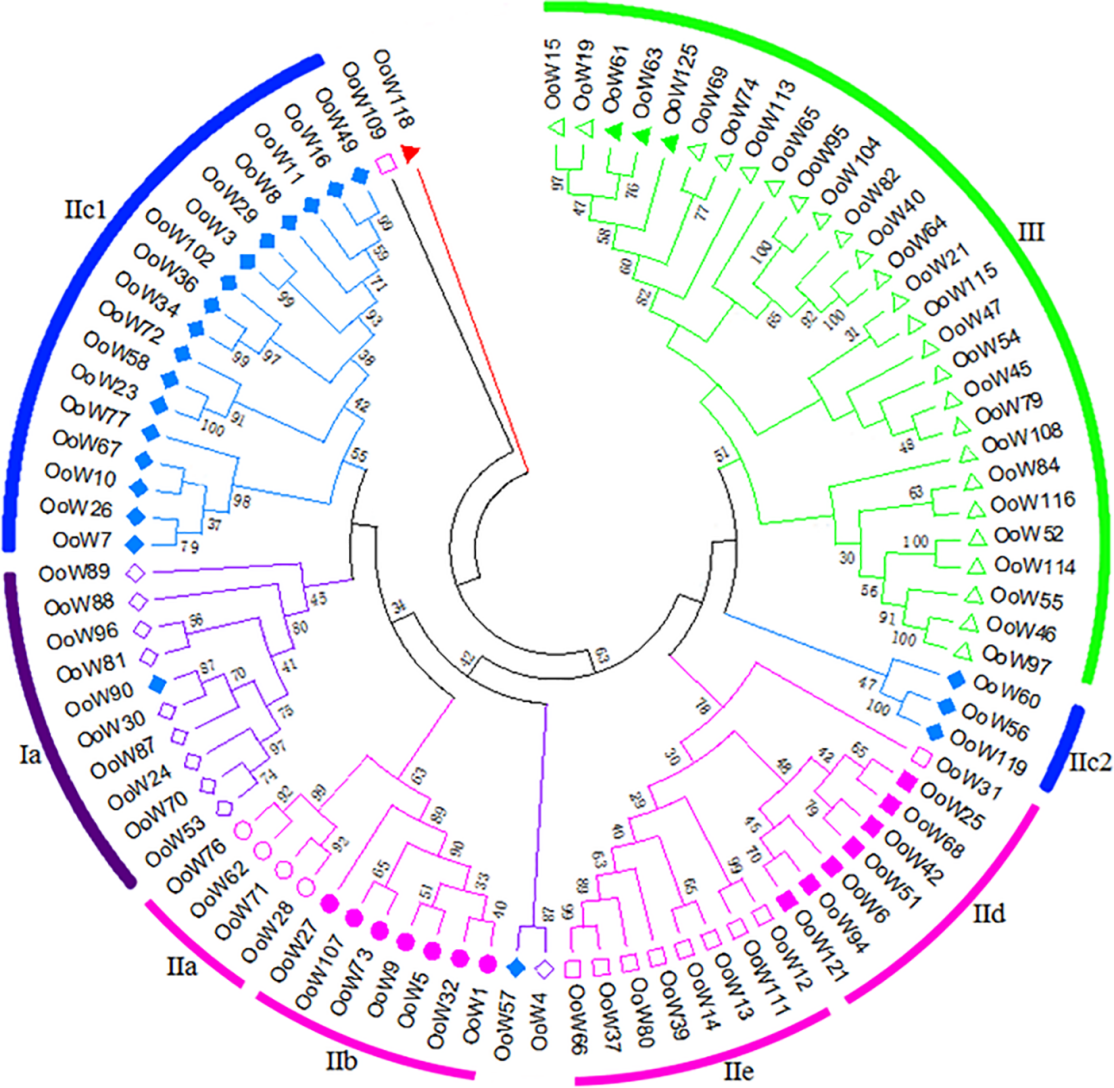
Phylogenetic analysis of full-length *OoWRKY* proteins. The amino acid sequences were aligned by MUSCLE in the MEGA6 using the default parameters. The NJ tree was shown by the results of 1,000 bootstrap replications. Bootstrap values were displayed with nodes. Group Ia: hollow diamond; Group Ib: filled triangle; Group IIa: circle; Group IIb: filled circle; Group IIc: filled diamond; group IId: filled square; Group IIe: square; Group III: triangle; OoWRKY118: red filled triangle.

The phylogenetic tree of *OoWRKY* domains might miss important information on the evolution of *OoWRKY* genes. Therefore, a NJ phylogenetic tree was constructed using the multiple sequence alignment of the full-length *OoWRKY* proteins (Fig 3, S3 File). The full-length proteins' phylogenetic tree was similar to the domain tree, but there were two main differences between two NJ trees. In the *OoWRKY* domains tree, the subgroup Ia *OoWRKY4* was clustered in the IaN and IaC clades, while *OoWRKY4* and *OoWRKY57* (IIc) were clustered into one clade in the NJ tree of full-length *OoWRKY* proteins, strongly supported by a high bootstrap value (87). In addition, subgroup IIe *OoWRKY 109* was clustered into IIe clade in *OoWRKY* domains tree, but fell out of IIe clade in the NJ tree of full-length *OoWRKY* proteins as *OoWRKY118*, supported by low bootstrap values (<20).

### WRKY motifs analysis of *OoWRKY* proteins

Most of *OoWRKY* proteins had a conserved WRKY motif, but some of them had a variant motif such as WKKY, WVKY, WRMC and WIKY (S2 Table). The sequence W(R/K) (K/R) Y is also recommended as the consensus sequence for the WRKY motif [27]. For example, the *OoWRKY* proteins had variant WRKY motifs of WKKY (*OoWRKY60*), WIKY (*OoWRKY63* and *OoWRKY125*), while *OoWRKY61* had two variant motifs of WVKY and WNKY. In addition, *OoWRKY118* had a variant motif of WRMC, which was different from W(R/K) (K/R) Y motifs. Although these *OoWRKY* proteins had a variant WRKY motif, they also contained the conserved zinc finger motifs.

Similarly, the WRKYGQK heptamer was present in the WRKY domain about 83% of *OoWRKY* proteins. WRKYGQK variants such as WVKYGQK (*OoWRKY61*), WNKYGQK (*OoWRKY61*) and WIKYDQK (*OoWRKY63*, *-125*) were found in subgroupⅠb *OoWRKY* proteins. The WRKYGKK (*OoWRKY7*, *-10*, *-26*, *-67* and *-77*) and WKKYGQK (*OoWRKY60*) motifs were found in subgroup IIc *OoWRKY* proteins. *OoWRKY46*, *-52*, *-55*, *-84*, *-97* and *-114* with WRKYGEK were found in group III. In addition, *OoWRKY118*, falling outside of group Ⅰ, II, and III, had a variant WRMCGQN that different from other WRKYGQK heptamer. The variant WRKYGQK of *OoWRKYs* was exhibited in S1 Fig with red box and S2 Table.

### Analysis of conserved motifs in *OoWRKY* proteins

**Fig 4:**
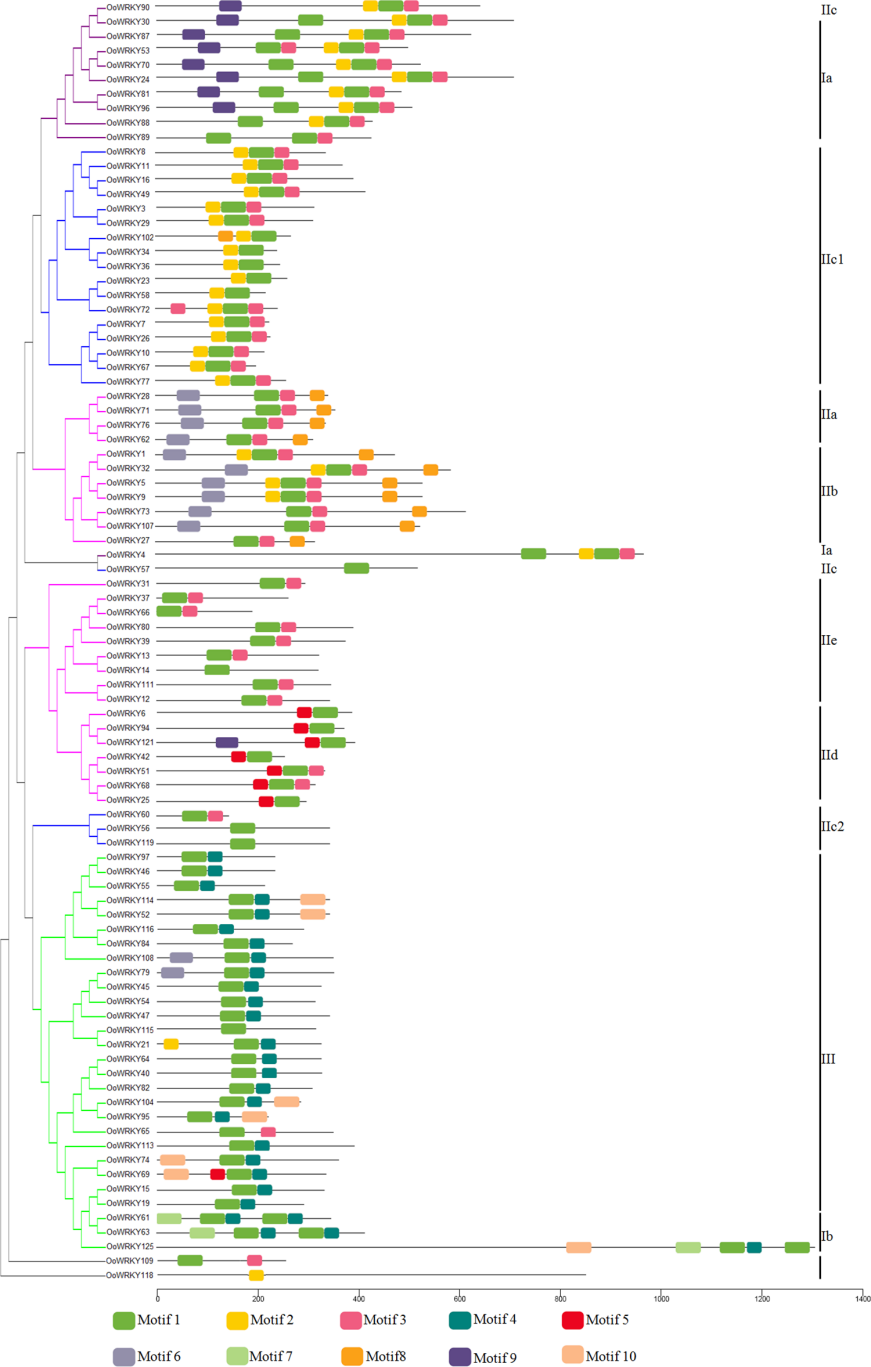
The conserved motifs arrangement of *OoWRKY* proteins based on their phylogentic relationships. The NJ tree was constructed from the amino acid sequences of *OoWRKYs* using MUSCLE and MEGA6 with 1,000 bootstrap replications. The conserved motifs in the *OoWRKY* proteins were identified by MEME. In total, ten conserved motifs were identified and showed in different colors.

MEME4.11.4 online software was used to analyze *OoWRKY* protein motifs, and Fig 4 and S2 Fig showed the ten conservative motifs in *OoWRKY* proteins. The same group of *OoWRKYs* had substantially consistent conserved motifs, which indicated there might be similar genetic functions. In subgroup Ⅰa, the C-terminal WRKY domains were consisted of motif 1, motif 2 and motif 3, while N-terminal domain only had motif 1. The WRKY domains of subgroup Ⅰb and group Ⅲ were composed of motif 1 and motif 4. The WRKY domains consisting of different motifs in subgroupⅠa and subgroup Ⅰb suggested that functional differentiation might occur in the groupⅠ*OoWRKY* genes. The WRKY domains of subgroups' Ⅱa, Ⅱb, Ⅱc, and Ⅱe were consisted of similar motifs (mainly motif 1 + motif 3 ± motif 2), while the WRKY domains of Ⅱd were mainly consitented of motif 1 and motif 5, indicating subgroups Ⅱd might have specific function.

### The expressed analysis of *OoWRKY* genes based on RNA-seq data

**Fig 5:**
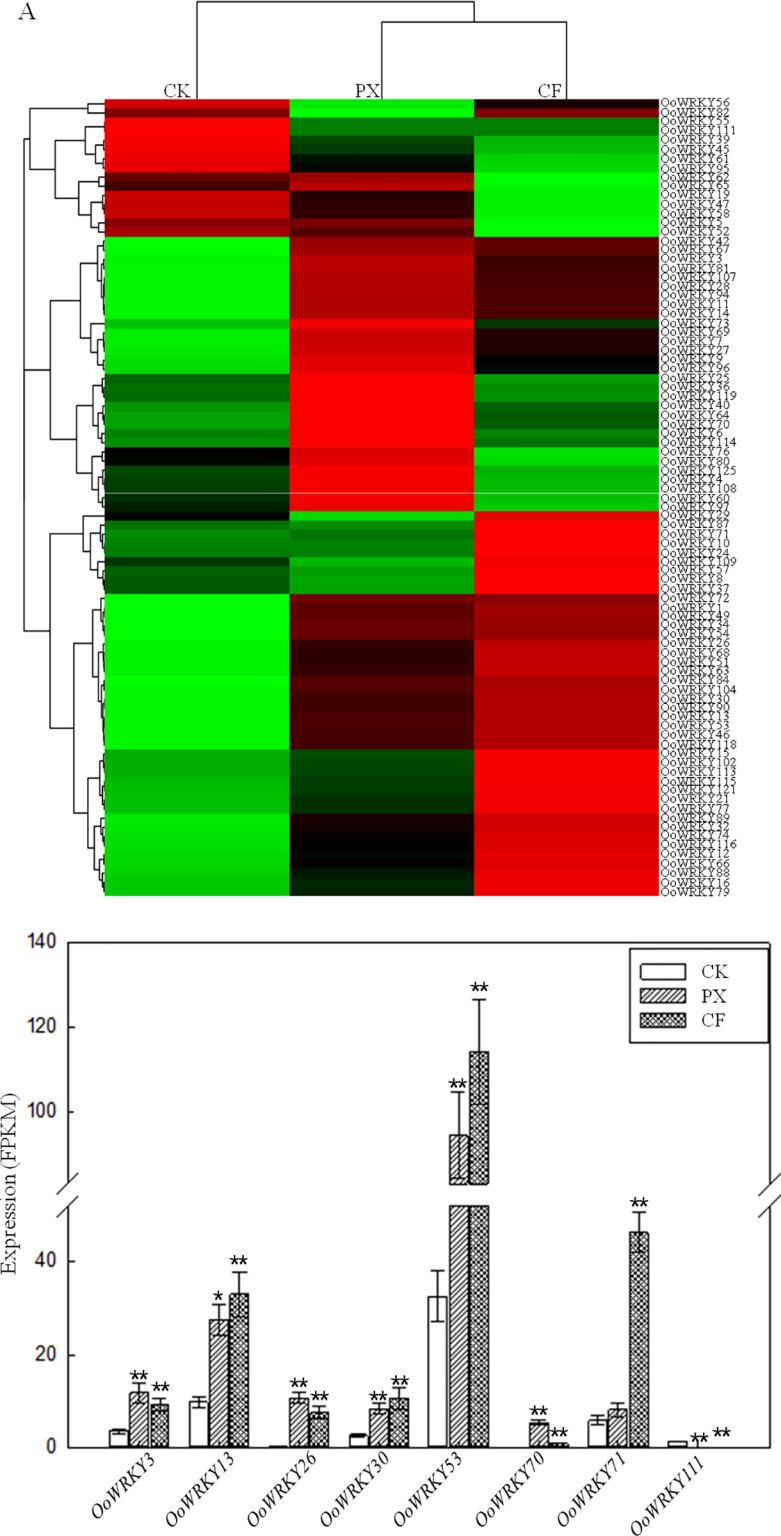
Expression profile analysis of *OoWRKY* genes by RNA-seq data. (A) The digital gene expression profiling of 89 *OoWRKY* genes in the CK, PX and CF transcript. The CK, PX and CF were dealt with ddH_2_O, *Xoo* strains PXO99 and C5 for 48 h respectively. Transcriptome data (Fragments Per Kilobase per Million fragments mapped, FPKM) was used to measure the expression levels of *OoWRKY* genes. (B) The selected significantly differential expression *OoWRKY* genes under PXO99 and C5 stress 48 h. One and two asterisks respectively indicated significant difference (< 2-fold, p<0.01) and extremely significant difference (> 2-fold, p<0.01) in gene expression in *O*. *officinalis* treated with PXO99 and C5 compared with the treated with ddH_2_O.

Based on the RNA-seq data analysis of *O*. *officinalis*, the expression level of 89 *OoWRKY* genes changed in the *O*. *officinalis* under *Xoo* stress at 48 h (Fig 5A), however, only eight *OoWRKY* genes were >2-fold up-regulated or down-regulated (p<0.01) (Fig 5B). Among these eight *OoWRKY* genes, six *OoWRKY* genes, including *OoWRKY3*, *OoWRKY13*, *OoWRKY26*, *OoWRKY30*, *OoWRKY53* and *OoWRKY70*, displayed increases in expression by >2-fold under PXO99 and C5 stress 48 h. In contrast, *OoWRKY111* was significantly down-regulated (>2-fold, p<0.01) under PXO99 and C5 stress 48 h. In addition, *OoWRKY71* displayed increases in expression by <2-fold (p<0.01) under PXO99 stress, while increases in expression by >2-fold (p<0.01) were observed under C5 stress. These eight genes displayed significant changes in expression might play important roles in PXO99 and C5 stress responses.

### Expression patterns of selected eight *OoWRKY* genes in response to *Xoo* strains PXO99 and C5

**Fig 6:**
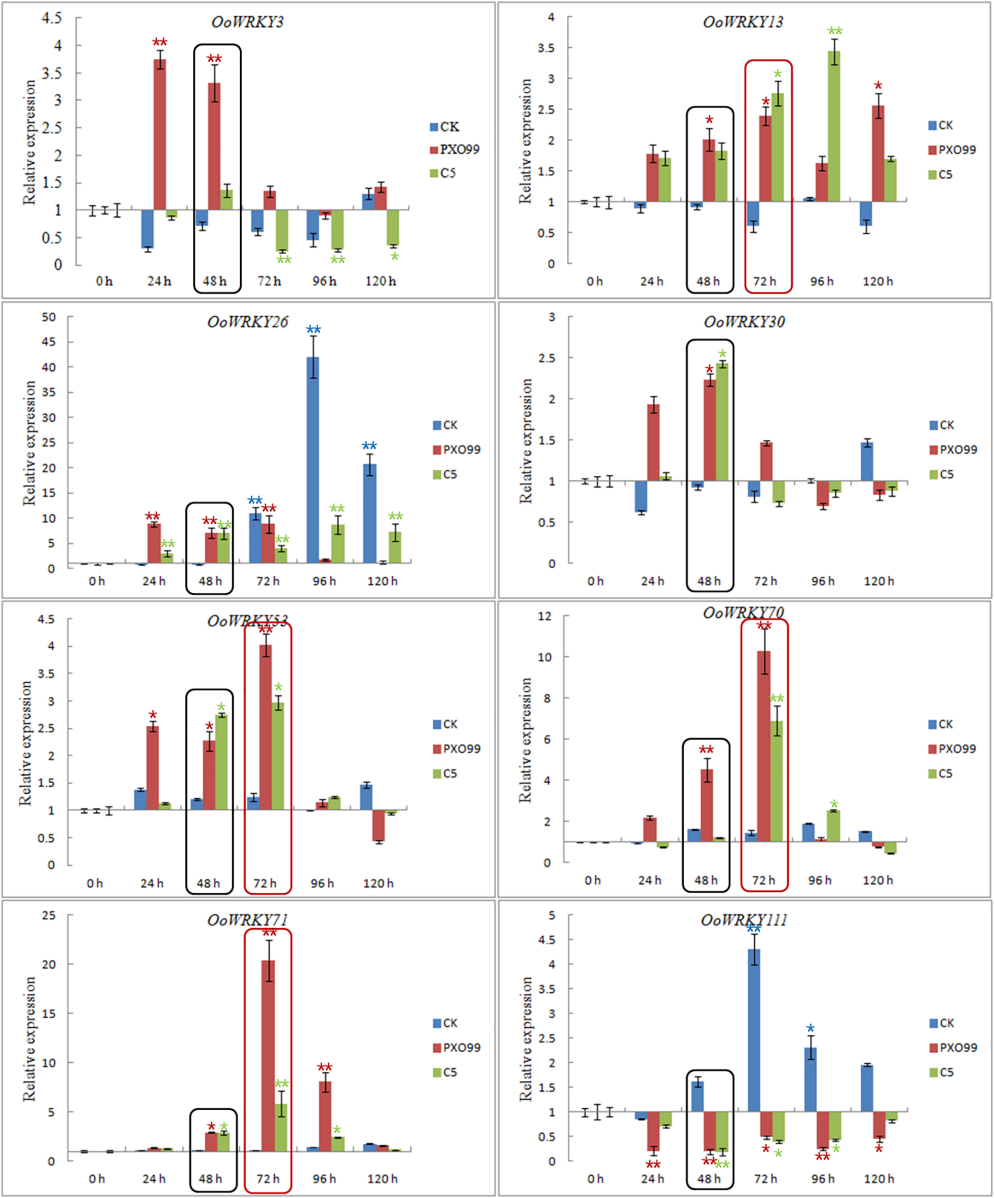
Expression of eight differentially expressed *OoWRKY* genes in response to PXO99 and C5. The relative expression levels of eight *OoWRKY* genes were measured by PXO99 and C5 stress 24 h, 48 h, 72 h, 96 h and 120 h compared with 0 h, respectively. Three independent replicates were used to generate each expression value. The error bars represented standard deviations. One and two asterisks respectively represented significantly different (p<0.05, n = 3) and extremely significant difference (p<0.01, n = 3) when assessed by Duncan's multiple range test.

In order to confirm some of the *OoWRKY* genes important for bacterial blight resistance, eight significantly differential expression *OoWRKY* genes were selected and their expression patterns were quantified by qRT-PCR at different time points under *Xoo* strains PXO99 and C5 stress. As shown in the Fig 6, seven *OoWRKY* genes were significantly un-regulated, while one *OoWRKY* displayed marked decreases in expression under *Xoo* stress (p<0.05). The expression levels of *OoWRKY13*, *OoWRKY53*, *OoWRKY70* and *OoWRKY71* were strongly increased under *Xoo* stress, especially at 72 h (Fig 6, red square frame).The expression of *OoWRKY71* under PXO99 stress 72 h was 20 times higher than that of 0 h. In contrast, the expression of *OoWRKY111* kept decreasing during PXO99 and C5 treatment, while displayed increases during the ddH_2_O treatment (CK). Interestingly, the expression patterns of *OoWRKY3* reversed in response to PXO99 and C5, which was significantly up-regulated under PXO99 stress 24 h and 48 h, while significantly down-regulated under C5 stress 72 h, 96 h and 120 h. *OoWRKY26* was significantly up-regulated under PXO99 and C5 stress, but the expression level was lower than that of treated with ddH_2_O (CK) from 72 h to 120 h. Except *OoWRKY13* and *OoWRKY111*, overall expressed tendency of other six *OoWRKY*s was significantly up-regulated under *Xoo* stress. The *OoWRKYs*, except *OoWRKY111*, were not significantly change at the different treatment time points in CK. The qRT-PCR results of the eight *OoWRKY*s under *Xoo* stress 48 h were consistent with the RNA-seq data (Fig 6, black square frame), which further demonstrated the reliability of our RNA-seq data. The expressed tendency of the same *OoWRKY* gene was almost unanimous under PXO99 and C5 stress, whether it was up-regulated or down-regulated.

### The organ specific expression of eight differentially expressed *OoWRKY* genes

**Fig 7:**
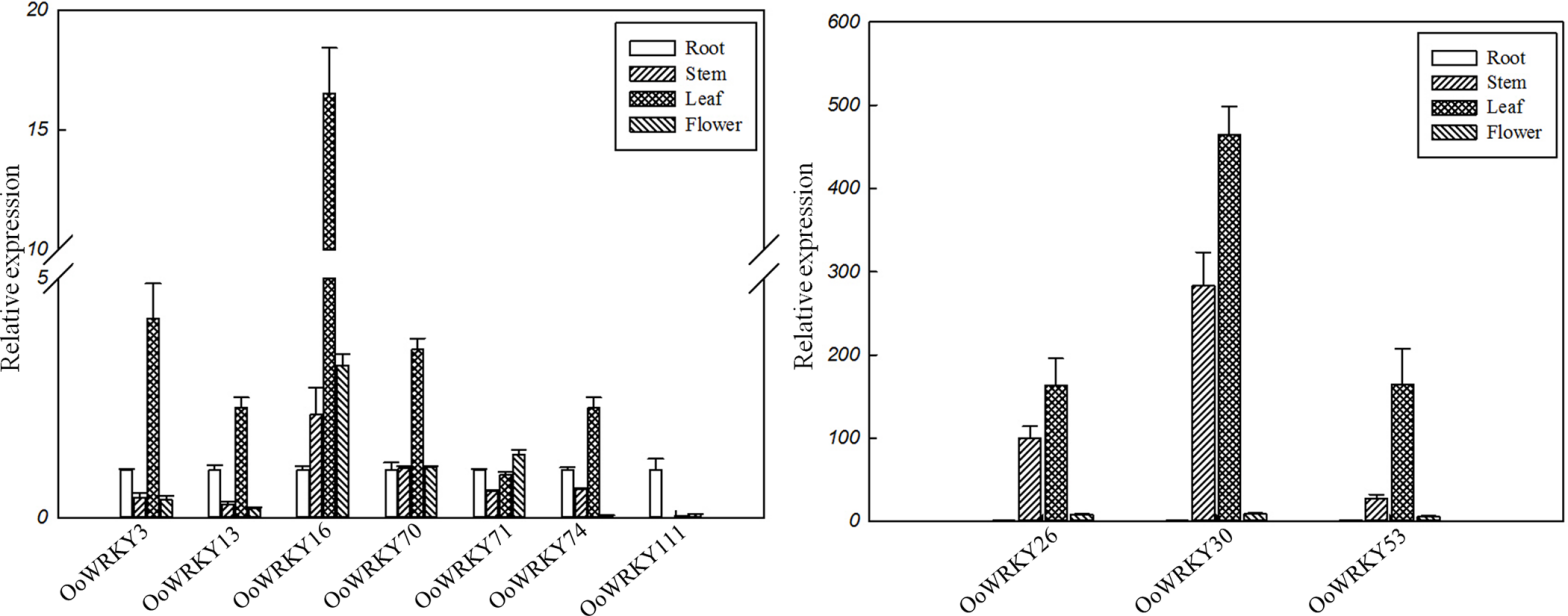
The organ-specific expression levels of eight differentially expressed *OoWRKY* genes in root, stem, leaf, and flower. The relative expression levels of eight *OoWRKY* genes were measured by the expression of stem, leaf and flower compared with root respectively. Three independent replicates were used to generate each expression value. The error bars represented standard deviations.

To confirm the functions of eight differentially expressed *OoWRKY* genes under PXO99 and C5 stress, we analyzed the expression of these *OoWRKY* genes in root, stem, leaf and flower of *O*. *officinalis* using qRT-PCR. The results showed that the eight *OoWRKY* genes were constitutive expression genes in root, stem, leaf and flower of *O*. *officinalis* (Fig 7). The expression levels of *OoWRKY71* and *OoWRKY111* were lower in leaf than that of in root, especially *OoWRKY71*, while the expression level of *OoWRKY111* was similar in stem, leaf and flower. The expression levels of other six *OoWRKY* genes were the highest in leaf, which was 3~400 times more than that of in root. The results suggested that *OoWRKY111* mainly functioned in root, while the other six *OoWRKY*s mainly functioned in leaf in the normal growth.

## Discussion

### The important of identification of *O*. *officinalis WRKY* genes

The *WRKY* transcription factors are one of the largest gene families, which involving in a wild variety of functions including plant development and stress response, and *WRKY* genes have been identified in many species, including kinds of plants, green algae, protozoa and slime mold [11]. Although *WRKY* genes of cultured rice and wild rice species *O*. *nivara* have many research reported [30], the *WRKY* genes in wild rice species of *O*. *officinalis* have not been studied. *O*. *officinalis* shows abundant genetic diversity and high resistance to bacterial blight [22–23, 31]. Thus, research on the *WRKY* genes of *O*. *officinalis* could not only provide a reference and theoretical basis for revealing the role of *O*. *officinalis WRKY* genes in the response mechanism of high resistance to bacterial blight, but also enrich the plant *WRKY* families.

In this study, a total of 89 *OoWRKY* genes were firstly identified from the transcriptome of *O*. *officinalis* by using an HMM model. The 89 *OoWRKY* genes were classified into groups' Ⅰ, Ⅱ and Ⅲ based on the number of WRKY domains and zinc finger motifs of WRKY domain sequences. Although *OoWRKY118* had a WRKY domain and a zinc finger motif, *OoWRKY118* could not be classified into group Ⅱ or Ⅲ for its WRKY domain and zinc finger type different from other *OoWKRY* genes. Comparison of the sequences of WRKY domains between *OoWRKY118* and *OsWRKY118* (LOC_Os08g09900.1), there were same variant WRMC and zinc finger motifs (S3 Fig), suggesting the difference sequence of *OoWRKY118* was not due to transcriptome assembling of *O*. *officinalis*. However, the *OoWRKY* numbers of identified from *O*. *officinalis* transcriptome database (670 Mb, 89 *WRKY* genes) was small compared with the *O*. *sativa* (440 Mb, 103 *WRKY* genes) and *Arabidopsis* (107 Mb, 72 *WRKY* genes), but our results provided a solid foundation for the study of the evolution and functions of *O*. *officinalis WRKY* genes.

### The evolution analysis of *O*. *officinalis WRKY* genes

The diversification of *WRKY*s classified into groups' Ⅰ, Ⅱ, Ⅲ and Ⅳ results from a long evolutionary history. There have been proposed several hypotheses to explain the evolution of *WRKY* genes. Hypothesis 1 proposes that subgroup Ia genes are the ancestor of other *WRKY* genes [4]. Hypothesis 2 propose that IIc-like genes are the common ancestor of subgroups Ia and IIc genes, and the other *WRKYs* evolve from subgroup Ia genes [32]. Hypothesis 3 proposes subgroup Ia genes are the most primitive ancestor and all other *WRKY*s evolve from the C-terminal domain of the subgroup Ia genes. Meanwhile, hypothesis 3 also suggests subgroups Ⅱ *WRKY* genes evolve directly from *WRKYs* that containing one WRKY domain [33]. Xu *et al* [30] propose that subgroup IIc genes are the original ancestor of *WRKY*s, and group I, Ⅲ and the other subgroup II *WRKYs* evolve from IIc genes.

Almost all of hypotheses could explain the evolution of *OoWRKYs*. Subgroup IIc *WRKY*s are ancestor of all *WRKY*s and display most diverse [4, 32]. The subgroup IIc *OoWRKY* genes were clustered into IIc1 and IIc2 clades in the phylogenetic analysis, indicating subgroup IIc *OoWRKY* genes were diverse. It was generally suggested that subgroup IIc *WRKY* genes evolved from C-terminal WRKY domain of subgroup Ia genes by losing the N-terminal WRKY domain. The subgroup IIc *OoWRKY* domain sequences were very similar to C-terminal *OoWRKY* domains of subgroup Ia, and they were many amino acids align identically (S4 Fig). In addition, subgroup IIc1 *OoWRKY* genes were clustered nearby the subgroup Ia. Subgroup IIc *OoWRKY90* was clustered into IaC clade (Fig 2), which indicated that *OoWRKY90* might evolve from the C-terminal domains of subgroup Ia. However, our results were also approved the hypothesis 2 that subgroup IIc genes were the ancestor of other *WRKY* genes. Most of subgroup IIc *OoWRKY* genes in IIc1 clade contained an RVE sequence in their zinc finger motifs, while most of group I *OoWRKYs* contained an HVE sequence in their zinc finger motifs (S4 Fig). *OoWRKY90* contained an HVE sequence in its zinc finger motif, which might be the reason *OoWRKY90* (IIc1) clustered into IaC in the phylogenetic tree. In addition, the subgroup IIc2 of *OoWRKY56* and *OoWRKY119* contained the same HVE sequence as IaC in their zinc finger motifs, indicating the subgroup Ia genes might evolve from subgroup IIc2 *WRKYs* by double WRKY domain. The close relationship between IIc and IaC indicated that the subgroup IIc (*OoWRKY90*) evolved from the C-terminal WRKY domain of the subgroup Ia, or subgroup Ia genes evolved from subgroup IIc *WRKYs* by double WRKY domain.

Hypotheses suggest the group Ⅲ *WRKY* genes evolve from the subgroup Ia and IId genes [32]. However, based on the zinc finger motifs of group Ⅲ and subgroup Ia *WRKYs*, Xu *et al* [30] propose group Ⅲ *WRKY*s diverge first from ancient subgroup IIc genes. They also propose if the group Ⅲ *WRKYs* evolve from subgroup Ia, then the zinc finger type of subgroup Ia *WRKYs* would be a C_2_HC [30]. However, all of the subgroup Ia *WRKYs* contain C_2_H_2_ zinc finger type, and all of group Ⅲ *WRKYs* contain C_2_HC zinc finger type [16, 30, 34]. Thus, based on the phylogenetic and zinc finger motif analysis of group Ⅲ *OoWRKY* genes in *O*. *officinalis*, we were more inclined to approve the hypothesis of Xu *et al* [30] that group Ⅲ *OoWRKY* genes diverged from subgroup IIc genes.

### The function of conserved motifs in *O*. *officinalis WRKY* genes

Although the WRKYGQK is highly conserved in most WRKY domains, frequently occurring variants of the core sequence are WRKYGKK and WRKYGEK [35]. Most of *OoWRKY* genes contained the WRKYGQK heptamer, while five subgroup IIc *OoWRKY* proteins (*OoWRKY7*, *-10*, *-26*, *-67* and *-77*) and six group Ⅲ *OoWRKY* proteins (*OoWRKY46*, *-52*, *-55*, *-84*, *-97* and *-114*) contained WRKYGKK and WRKYGEK, respectively. The variant WRKYGKK and its functions have also been reported in most plant species [35–37]. The WRKY domain-DNA interactions can activate the plant development and defense [38], but the WRKYGKK variant reduces DNA-binding to the W-box, which reported losing the ability of binding to W-box domain in soybean [39]. In our study, the expression level of *OoWRKY26* was significantly up-regulated before 72 h under PXO99 and C5 stress, but the *OoWRKY26* expression level under *Xoo* stress after 72 h was lower than that in CK (ddH_2_O treatment), suggesting that the regulated expression of *OoWRKY26* under *Xoo* stress after 72 h might be mainly due to mechanical injury during inoculation treatment. PXO99 and C5 induced the expression of *OoWRKY26* in *O*. *officinalis*, while *WRKY26* enhances the tolerance of plants to high temperature stress regulated in *Arabidopsis thaliana* [40]. Therefore, *OoWRKY26*, containing WRKYGKK heptamer, might be simultaneously involved in biotic and abiotic stress responses in *O*. *officinalis*.

Identification of conserved domains on *OoWRKY* genes could help elucidate their functions in *O*. *officinalis*. Extra domains were found in *OoWRKY* proteins by using the MOTIF search program (S3 Table). Interestingly, subgroup Ib *OoWRKY125* contained not only two WRKY domains but also an NB-ARC domain. NB-ARC domain, sharing by plant resistance gene products and regulators, functioned as a signaling motif in plant resistance [41]. The genes that contain both WRKY and NB-ARC domains are classified as *RWRKY* genes, which are also found in *OsRWRKY1* and *OsRWRKY2* [41]. NB-ARC domain has also been identified in *Arabidopsis thaliana*, *Fragaria vesca*, *Glycine max*, *Sorghum bicolor*, *Setaria italica*, and *Theobroma cacao* [33, 41]. NACHT domain, an evolutionary conserved protein domain associated with apoptosis and MHC transcriptional activation [42], was also found in *OoWRKY125*. Therefore, we conjectured *OoWRKY125* might play an important role in the immune response of *O*. *officinalis*. Plant_zn_clust only occured in subgroup IId *OoWRKYs*, indicating these genes possessed special function. FLYWCH zinc finger domains were found in subgroup IId *OoWRKY6*, subgroup IIe *OoWRKY39* and group III *OoWRKY40*, which suggested they might play a role in DNA-RNA binding or protein-protein interactions [43]. FAR1 domain, involving in the phyA-signaling pathway [44], was found in subgroup Ⅰa *OoWRKY24* and subgroup IIc *OoWRKY7*, *-26*, *-67*. It suggested that these *OoWRKYs* with FAR1 domain might function in the phytochrome signaling pathway in *O*. *officinalis*. Mito_fiss_reg domain, relating to mitochondrial fission [45], was presented in subgroup IIa *OoWRKY71*. *OsWRKY71*, involving in elicitor-induced defense responses in rice [46], also contains the Mito_fiss_reg, and the subgroup IIa *OsWRKY* transcription factors are reported to mediate rice innate immunity [47]. Therefore, the Mito_fiss_reg domain could help to explore the subgroup IIa *OoWRKYs*' functional mechanism in *O*. *officinalis*. These extra domains could help elucidate the function and evolutionary relationships of *OoWRKY* genes.

### The eight significantly differential expression *O*. *officinalis WRKY*s in response to bacterial blight resistance

*WRKY* transcription factor plays an important role in the defense mechanism of plant disease resistance as a positive regulator or negative regulator [8]. There have been identified more than 100 *WRKY* genes from *O*. *sativa*, of which 35 *WRKY* genes play an important role in disease resistance, such as *OsWRKY3*, *OsWRKY13*, *OsWRKY28*, *OsWRKY30*, *OsWRKY31*, *OsWRKY45*, *OsWRKY53*, *OsWRKY70*, *OsWRKY71*, they play an important regulatory role in disease resistance-related pathways of bacterial blight, rice blast and sheath blight [17]. Although many plant *WRKY* genes associated with disease resistance have been reported, there is no any research report about resistance to disease-related *WRKY* genes in *O*. *officinalis* due to lacking of its genomic information. Based on the expression levels of 89 *OoWRKY* genes of *O*. *officinalis* under PXO99 and C5 stress, we selected eight *OoWRKY* genes of marker changed in expression under *Xoo* stress.

Liu *et al* [48] reveal that *OsWRKY3*, as a transcriptional activator in SA-dependent or JA-dependent on disease-resistant signal cascades, is down-regulated at 12 h and up-regulated at 48 h under *Xoo* stress. We found the expression patterns of *OoWRKY3* were different in response to PXO99 and C5. PXO99 induced the expression of *OoWRKY3*, whereas C5 inhibited its expression. The expression level of *OoWRKY3* was significantly up-regulated under PXO99 stress 24 h and 48 h, while significantly down-regulated under C5 stress from 72 h to 120 h. Although the *OoWRKY3* expression was significantly up-regulated under PXO99 as reported by Liu *et al* [48], Liu *et al* do not clear the strains of *Xoo*. *OoWRKY3* had different expression patterns in response to different *Xoo* strains, and the expression of *OoWRKY3* was the highest in leaf, which indicated it mainly functioned in leaf. Therefore, we suggested *OoWRKY3* may regulate different defense genes expression in response to different *Xoo* strains by self expression level. *OsWRKY13* directly or indirectly mediates disease resistance to bacterial blight and fungal blast through activation of SA-dependent pathways, suppression of JA-dependent pathways [49]. In addition, *OsWRKY13* also activates the flavonoid biosynthesis pathway, which enhances the biosynthesis of antimicrobial flavonoid phytoalexins [50]. The expression level of *OoWRKY13* was 2~3 times higher under PXO99 and C5 stress than that of ddH_2_O treated (CK), indicating that the increased expression of *OoWRKY13* was induced by PXO99 and C5. The up-regulated expression of *OoWRKY13* also activated the flavonoid biosynthesis pathway and increased the accumulation of flavonoid. Flavonoid can accumulate as phytoalexins in plant to enhance plants resistance to pathogen [51]. There is report that the flavonoid phytoalexin in resistant rice contribute to blast resistance [52]. Therefore, *OoWRKY13* might enhance the resistance to PXO99 and C5 in *O*. *officinalis* by activating the flavonoid biosynthesis pathway and increasing the accumulation of flavonoid, indicating *OoWRKY13* might be a new bacterial blight resistance gene of *O*. *officinalis*.

*OsWRKY30* is constitutive expression gene in the root and leaf of *O*. *sativa*, and the expression of *OsWRKY30* induces rapidly by *Magnaporthe grisea*, SA and JA [53]. Ramamoorthy *et al* [54] suggest the expression level of *OoWRKY30* cannot be observed in the root and leaf of normal growth. Over-expression of *OsWRKY30* enhances the resistance of transgenic rice plants to fungal blast [55]. Han *et al* [56] prove that *OsWRKY30* mediates disease resistance to bacterial blight as a positive regulator through the SA signaling pathway. The expression level of *OoWRKY30* displayed increases only under PXO99 and C5 stress 48 h, indicating the affection of PXO99 and C5 on *OoWRKY30* was transient. The expression level of *OoWRKY30* was very high in stem and leaf of normal growth in *O*. *officinalis*, which was 300~500 times more than that in root. As a high and constitutive expression gene in leaf, we speculated *OoWRKY30* would play an important role in maintaining the normal growth of leaf, and its high expression level was enough to regulate the resistance-related pathway in *O*. *officinalis* without increasing its expression under PXO99 and C5 stress.

*OsWRKY53* is a transcriptional activator in plant defense, which triggers the expression of other transcription factors of the same or different family of genes that involved in disease resistance-related metabolic pathways [18]. Over-expression of *OsWRKY53* enhances the resistance of transgenic rice plants to bacterial blight and fungal blast [18]. The *OoWRKY53* expression level was the highest in the normal growth leaf of *O*. *officinalis* among eight *OoWRKY* genes, and its expression significantly increased under PXO99 and C5 stress, indicating that *OoWRKY53* not only played a role in the normal growth of leaf, but also played an important role in disease resistance to PXO99 and C5 in *O*. *officinalis*.

*OsWRKY71* plays an important role in rice defense response. Over-expression of *OsWRKY71* enhances the resistance of transgenic rice to the *Xoo* strain 13571 [19]. The sequences of WRKY function domains between *OsWRKY71* and *OoWRKY71* were completely consistent (S5 Fig showed with black box). Therefore, *OoWRKY71* also had the resistance to *Xoo* strain 13571 as *OsWRKY71*. In *O*. *officinalis*, the expression of *OoWRKY71* was extremely low in the normal growth of leaf, but the expression level significantly increased under PXO99 and C5 stress. In view of the analysis of *OoWRKY71* expression trends under PXO99 and C5 stress, we suggested the up-regulated expression of *OoWRKY71* would enhance the resistance of *O*. *officinalis* to the PXO99 and C5. So, we speculated *OoWRKY71* not only had resistance to *Xoo* strain 13571 as *OsWRKY71* but also resistance to strains PXO99 and C5, and the bacterial blight resistance of *OoWRKY71* might be stronger than *OsWRKY71* due to *OoWRKY71's* strong response to the PXO99 and C5. *OoWRKY71* should have an important effect on bacterial blight resistance in *O*. *officinalis*.

There are no reports about the function of *WRKY26* and *WRKY111* genes in rice. *WRKY26*, regulating positively in the signaling pathway of ethylene activation and heat shock protein, enhances the tolerance of plants to high temperature stress [40]. *OoWRKY26* displayed increases in expression under PXO99 and C5 stress 24 h, 48 h, 72 h, but the expression level of *OoWRKY26* under *Xoo* stress from 72 h to 120 h was lower than that ddH_2_O treated (CK), which indicated that the expression chances of *OoWRKY26* under *Xoo* stress starting from 72 h might be due to mechanical injury during inoculation treatment. *Xoo* infects the rice from the wound of leaf, stem, or root, then propagate and transmit in the vascular bundle. The expression of *OoWRKY111* continued to decrease significantly under PXO99 and C5 stress, suggesting *OoWRKY111* acted as a negative regulator mediated resistance-related pathways of genes in *O*. *officinalis*. The expression of *OoWRKY111* was higher in root than that in leaf, stem, and flower. Therefore, we speculated the *OoWRKY111* would play an important role in defense of the pathogen infection in root.

It can predict gene function by analyzing the expression of the gene in certain condition and the organism. The eight *OoWRKY* genes displaying significant change in expression under *Xoo* strains PXO99 and C5 stress might play important roles in *Xoo* stress responses in *O*. *officinalis*. The expressed tendency of the same *OoWRKY* gene was almost unanimous under PXO99 and C5 stress, whether it was up-regulated or down-regulated, but the expression levels were different. The *O*. *officinalis* had strong resistance to C5, but its resistance to PXO99 was weak (unpulished data). The differentially expressed levels of *OoWRKY* genes in response to PXO99 and C5 might be one of the reasons for the different resistance of *O*. *officinalis* to the PXO99 and C5. In this study, the marked expression changes of eight *OoWRKYs* under *Xoo* stress and the specific expression in root, stem, leaf, and flower supplemented the results of existing studies, and analyzed the function of *OoWRKY* genes in response to PXO99 and C5 in *O*. *officinalis*, which provided a theoretical basis of studying the *WRKY* genes in mechanism of *O*. *officinalis*-*Xoo*.

## Conclusion

We identified 89 *O*. *officinalis WRKY* genes and focused on those involved in response to *Xoo* stress. The classification, structure, and evolutionary characteristics of *O*. *officinalis WRKY* genes were analyzed. *OoWRKY* genes expression patterns revealed that eight *OoWRKY* genes displaying significant changes in expression might play an important role in PXO99 and C5 stress responses in *O*.*officinalis*. In addition, tissue expression profiles showed that the eight *OoWRKY* genes also played different roles in *O*. *officinalis* development and exhibit differential expression level in different tissues. *OoWRKY13* might enhance the resistance to PXO99 and C5 in *O*. *officinalis* by activating the flavonoid biosynthesis pathway and increasing the accumulation of flavonoid, and might be a new bacterial blight resistance gene of *O*. *officinalis*. *OoWRKY71* had resistance to *Xoo* strain 13571 as *OsWRKY71*; even the resistance of *OoWRKY71* might be stronger than *OsWRKY71* due to *OoWRKY71's* strong response to the strains PXO99 and C5. In conclusion, our study established a structural and functional framework to investigate *O*. *officinalis WRKY* genes by the RNA-seq under the condition of lacking of *O*. *officinalis* genome. This study provided a solid foundation for the study of the evolution and the functions of *O*. *officinalis WRKY* genes in response to *Xoo* stress.

## Supporting information

S1 Fig The multiple alignments of OoWRKY domain peptide. Red square frames were WRKYGQK variants. Black lines represented WRKY motifs; Black solid triangle and black background indicated cysteine and histidine residues of zinc finger motifs. The red square frames represented the WRKYGQK variants.
(TIF)

S2 Fig The motif structure of ten motifs in *OoWRKYs*. (TIF)

S3 Fig The amino acid alignment of the WRKY domain and zinc finger motif in OoWRKY118. Black line represented WRKY motifs; Black solid triangle indicated cysteine and histidine residues of zinc finger motifs.
(TIF)

S4 Fig The amino acid alignment of subgroup Ia C-terminal OoWRKY domains and subgroup IIc OoWRKY domains. (TIF)

S5 Fig The amino acid alignments between *OoWRKY71* and *OsWRKY71*. The amino acids of black frame were the WRKY domains.
(TIF)

S1 Table qRT-PCR primers of significantly different expression *OoWRKYs*. (XLSX)

S2 Table *WRKY* genes identified in *O*. *officinalis* and their orthologs in *O*. *sativa sbb japonica*. (XLSX)

S3 Table Extra domains found in *OoWRKY* proteins. (XLSX)

S1 File The amino acid sequences of *WRKY* in *O*. *officinalis*. The red backgrounds were the amino acid sequences of conserved WRKY and zinc finger motif.
(PDF)

S2 File Aligment of OoWRKY domain amino acid sequences. The alignment was performed by MUSCLE in the MEGA6.
(MEG)

S3 File Aligment of OoWRKY full length amino acid. The alignment was performed by MUSCLE in the MEGA6.
(MEG)
