# Supplementary figures and images for: Transcriptome analysis of WRKY gene family in Oryza officinalis Wall ex Watt and WRKY genes involved in responses to Xanthomonas oryzae pv. oryzae stress
Source: PLoS One. 2017 Nov 30;12(11):e0188742. doi: 10.1371/journal.pone.0188742 (PMC5708796; doi:10.1371/journal.pone.0188742)

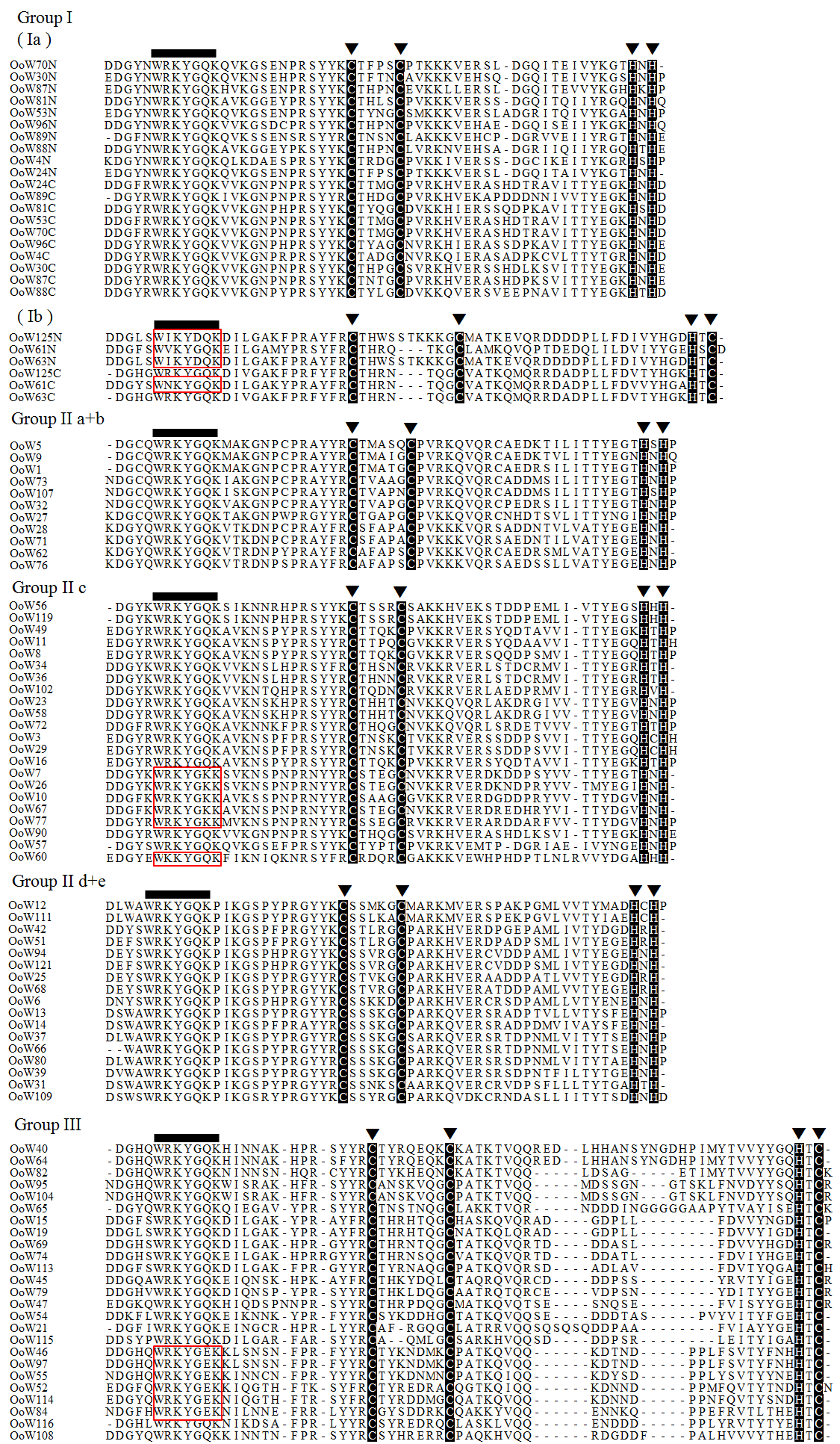

Supplement: S1 Fig — Red square frames were WRKYGQK variants. Black lines represented WRKY motifs; Black solid triangle and black background indicated cysteine and histidine residues of zinc finger motifs. The red square frames represented the WRKYGQK variants. (TIF) [file pone.0188742.s001.tif]

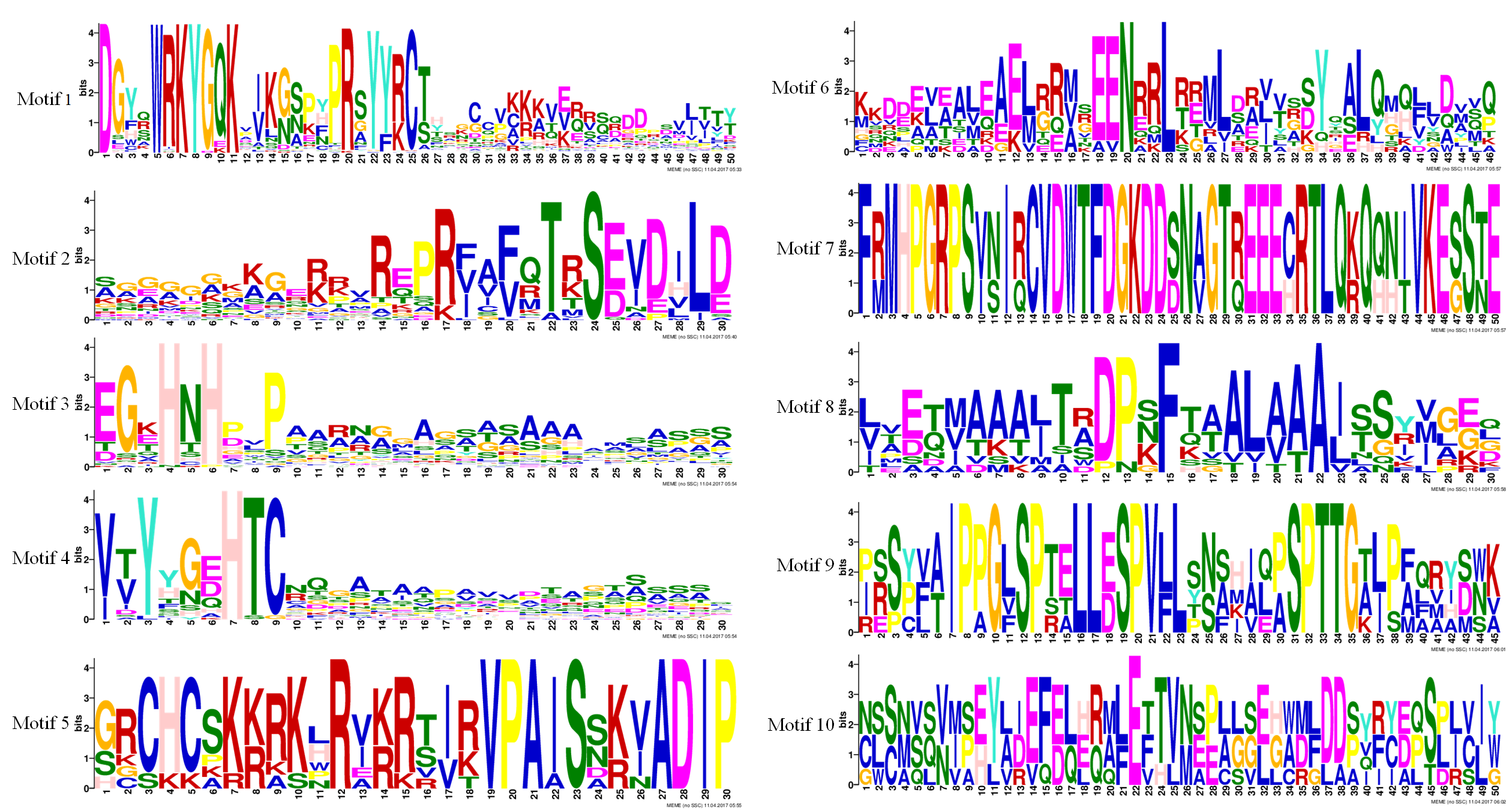

Supplement: S2 Fig — (TIF) [file pone.0188742.s002.tif]

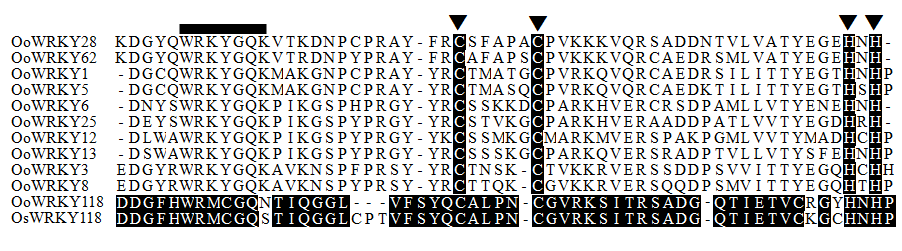

Supplement: S3 Fig — Black line represented WRKY motifs; Black solid triangle indicated cysteine and histidine residues of zinc finger motifs. (TIF) [file pone.0188742.s003.tif]

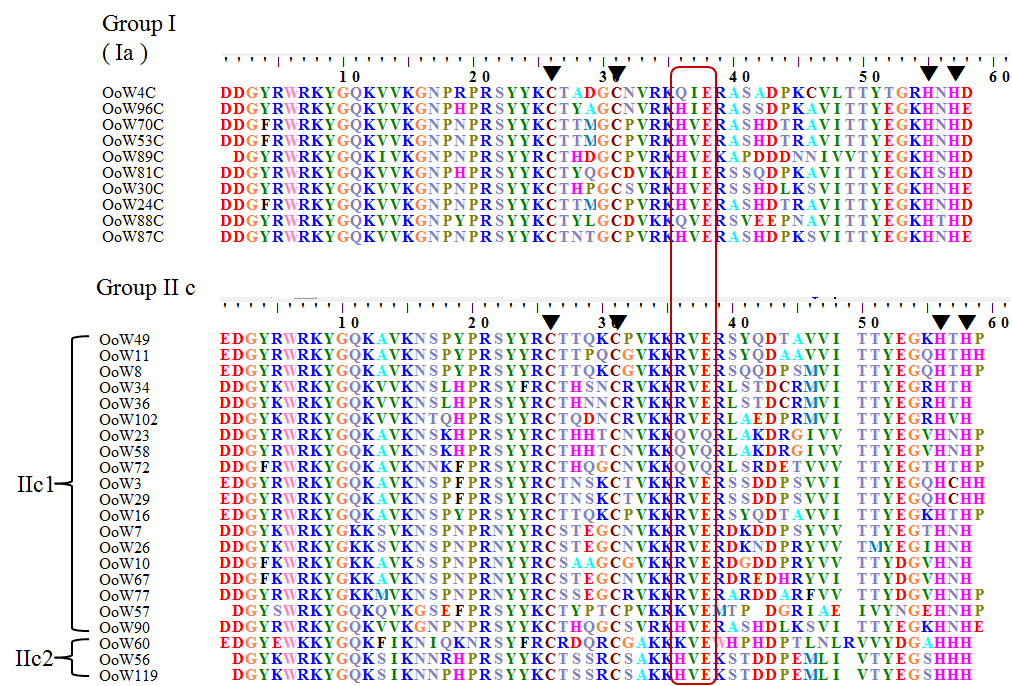

Supplement: S4 Fig — (TIF) [file pone.0188742.s004.tif]

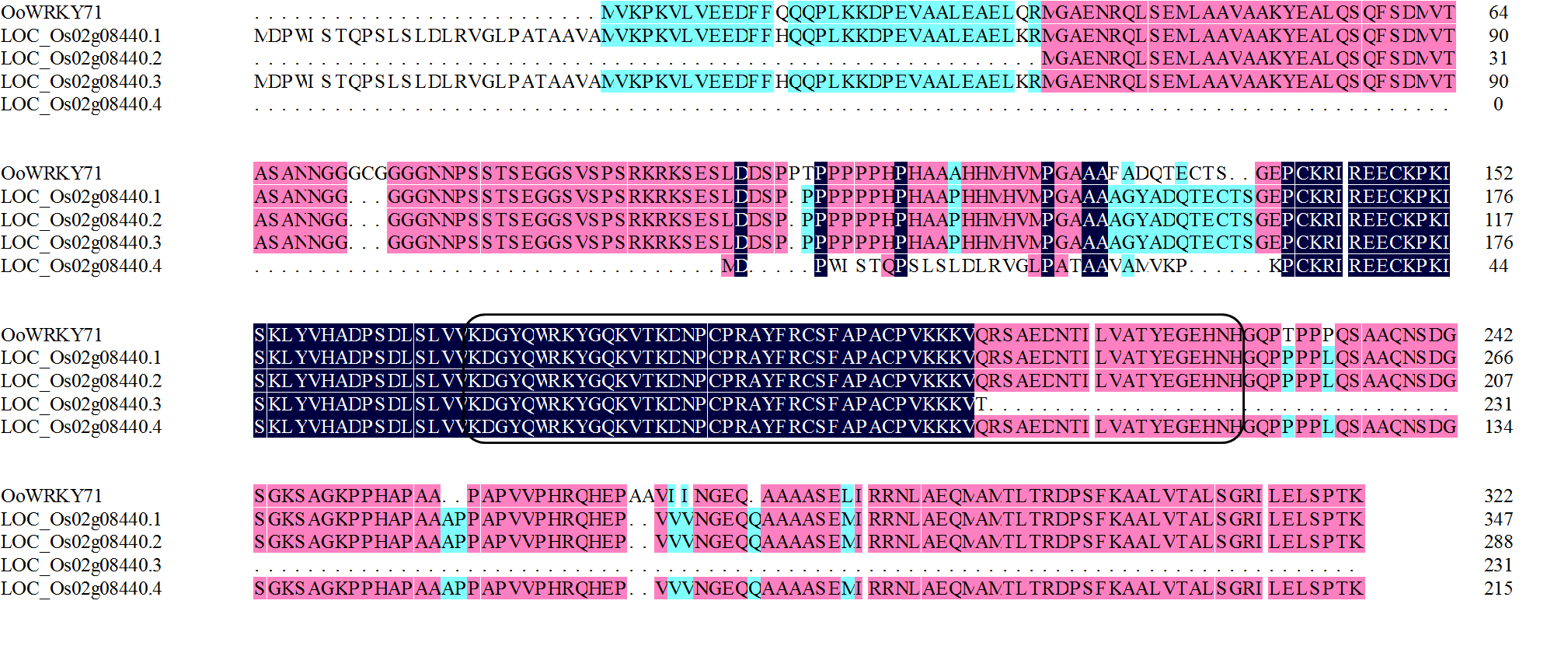

Supplement: S5 Fig — The amino acids of black frame were the WRKY domains. (TIF) [file pone.0188742.s005.tif]
