# Supplementary material for: Transcriptome analysis of WRKY gene family in Oryza officinalis Wall ex Watt and WRKY genes involved in responses to Xanthomonas oryzae pv. oryzae stress
Source: PLoS One. 2017 Nov 30;12(11):e0188742. doi: 10.1371/journal.pone.0188742 (PMC5708796; doi:10.1371/journal.pone.0188742)
Supplement: S1 File — The red backgrounds were the amino acid sequences of conserved WRKY and zinc finger motif. (PDF) [file pone.0188742.s009.pdf]

>OoW1
MDGSLDIYALSLSCACDVLQLAAMQAELGRMNEENQRLRGMLTQVTSSYQALQMHLVALMQQRPQMMQP
PTQPQPPPAHQDGKAEGAVVPRQFLDLGPSGAGGEAAEEPSNSSTEAGSPRRSSSTGNKDQERGDSLDAPST
AAAWLPGRAMAPQMGGAASKGHDQQAQEANMRKARVSVRARSEAPIIADGCQWRKYGQKMAKGNPCP
RAYYRCTMATGCPVRKQVQRCAEDRSILITTYEGTHNHPLPPAAMAMASTTSAAASMLLSGSMPSADGAAGI
MSSNFLARTVLPCSSSMATISASAPFPTVTLDLTHAPPGAPNAVPLNAARPAGPAPQFQVPLPGGGMAPAFAV
PPQVLYNQSKFSGLQMSSDSAEAAAAAQFAQPRPPIGQLPGPLSDTVSAAAAAITADPNFTVALAAAITSIIGGQ
HAAAAGNSNANNTNTNTTSNTNNTSSNNTTSNNTNSETQ*
>OoW3
GGEWSLFDGDAFAEYSSAVLAELGGWAAGAGGGGMMIPVLDLPEDVMGAAAARSEEEAPARSGDGGAAAS
SSSSGEPAAPENADSGKQPAIAEAAPAAAATAKKGQKRARQPRFAFMTKSEIDHLEDGYRWRKYGQKAVKNSP
FPRSYYRCTNSKCTVKKRVERSSDDPSVVITTYEGQHCHHTASFQRGVGGAAVAAHIHGAAAVALAEQMSAFV
SAPPPPAPPQQHMLYSLPRLHPPSSSETVVSSMPTTSLQELNNSEGLQRPSYSPQAAVTIAQPPPSPSVPPAVSF
DKGLLDDIVPPGVRLG*
>OoW4
MAEALVAGLRLAASAVARPQSRRRRGRLPDGYCAARVPQLRRGRLCARAAVAGPPEVDEDEAMSIDNLRRFF
DVNVGKWNGAFYQFDAHGRVLQGISTRLSVSTYGEDDLISLLQSLYIKQASSQISFVDEEDSEEWVEYKIKETNM
FTVDKYQQIGFFREEKAFALRYQTAGMLETVLRAGVLGEDDTGEESPKNLKIPSRKPSIVCENCLYSLEGNGRVRA
FHIMDPKGVLDMLIIFHEKQGSEVPLIYSSDDADITNNDRIAPLLGRWEGRSVTKRSGVYGATLSEADTVVLLEK
DRSGQLILDNMSTKSGTSTTTTVHWTGSANNNLLRFDGGYEMTLLPGGMYMGYPSDIGKIVNDLDSFHLEFC
WMESPGKRQRLVRTYDSAGLAVSSTYFFETKRLSSLKPHHHLRRRVGDHGRTPDLSNANRVAGEGEGGDRFR
RRRVAAVTAAAIDRAKSPEEGDSFRWFWKYSAQAVGASQSNPNTSRSDPLSWSAAVVVIDREALPLHKQRKLT
RAAAVAMADRRRSDGGGGMQQQPFTSPGQERVFDGGGVPGQVAPYGSDFDQSSYMALLAAGAVGVQPTT
APWAVEEDVAAAPPGISLAPQFSMANYAPPPSYQHPATLVSPPLAAGLHPYPPYLHGVDAPPPQWPPRPAPSP
SSLQPPPPNFTVLAAAAPPHEQHHSMQQLLLRAAAFGGGMHAVPTAAPAAATIEQPAKDGYNWRKYGQKQL
KDAESPRSYYKCTRDGCPVKKIVERSSDGCIKEITYKGRHSHPRPLEPRRGSSSSGMGAMAAGDHNAGAAADD
AAVVDEDDHSDDDTLHEDDDGEEEGHDSRGADGEVGQRVVRKPKIILQTRSEVDLLDDGYRWRKYGQKVVK
GNPRPRSYYKCTADGCNVRKQIERASADPKCVLTTYTGRHNHDPPGRPAGAAAAASANLQMPGPAAMSLAG
GGARQMKEET*
>OoW5
MEMMVQKQRNEEEEERGLLIGREIKEVDFFSAAAGAGGGRRDVDDDGAVVLRADGISSHAGFMVSTALDLLT
AVNDGDEEKKGEYSSDHQSKMDAAATTVEGELRQAGEENRRLRRRLEELTTSYGALYHQLVQAQQLHTKQQV
AMAGVQLLDALAASPASHRRRAEAAAALDGDRTDSDGGEADKNVSPSLGSKRPATTLTRLTPESGGGENNAG
GEQAPAAEMAPCRKARVSVRARSEAPMISDGCQWRKYGQKMAKGNPCPRAYYRCTMASQCPVRKQVQRC
AEDKTILITTYEGTHSHPLPPAAAAMAKTTSAAAAMLLSGPAVSRDALFGHHGAVVAAPPFFHHPYGGAAGGS
AATTTMATLSASAPFPTITLDLTQPPPTTATGAAGMLHRPQAFPSLPFSMYGGAGAGFPGSHRHVVLPPPSPAS
SGTSPMGARERSVLETMTAAITRDPNFTTALTAALSSIMAGDAQAQTPPRGGSAATGDAAGDINGGGGAHHP
TAGARAAATQPCSTSPT*
>OoW6
MDGVEEANMAAVESSKKLVAILSQSGDPFRLMAAVAETDEAVSRFGKVVTILSNRVGHARARLGKRRSSPPVD
LGCLMDHPLAAAAASCPAPTNGCLHFSTTTSPAPPSPATAAAAASSAAKITPAVVDRSLFLQTTLLDLNSCGAPAP
APAASMAASAKNSSKLGAAPAPMVNSSSSANHIQFQPPMKQQMKSFQFEQKPVSDKFHIEMPRSGGGGKEV
ISFSFDNSVCTSSAATSFFTSISSQLISMSDATTNSAAAAPTKKPSSCARKADDDAGGKCHCPKKKKPREKKVVTV
PAISDKVADIPSDNYSWRKYGQKPIKGSPHPRGYYRCSSKKDCPARKHVERCRSDPAMLLVTYENEHNHAQPLD

ISLVQQATANPQA*
>OoW7
MAAVGAHAAVYHHPVSGLSAPAGDAYSMSSYFSHGGSSTSSSASSFSAALTAATPPLPDPSGAQFDISEFFFDD
APPAAVFNGAPTAVLPDGGAASAAAHATRSAAEAVPAPAAVERPRTERIAFRTKSEIEILDDGYKWRKYGKKSVK
NSPNPRNYYRCSTEGCNVKKRVERDKDDPSYVVTTYEGTHNHVSPSTVYYASQDAASGRFFVAGTQPPGSLN*
>OoW8
MSGPGGGGHGLYEDHPAAGFVPFDHDDDVVDSFFFGTSASGGAGAGAGAGGDDGVGLITPYSSITDYLQGFL
QDPVYASSPMGDAAVKHESVVDHASQAGGAAAPATPNSSSVLSSSSEAAGGDDLRRCKRGRRPEDDDEEIDD
EGSAVQSCKTNKMKNKKGEKKEREPRVAFMTKSEVDHLEDGYRWRKYGQKAVKNSPYPRSYYRCTTQKCGVK
KRVERSQQDPSMVITTYEGQHTHPSPVSYHVHRQQGLMSARAVMAGAGYPFGAPPPPPLLGFDEALAARVT
MNQQQLGFVPSIHAARAPPTMPPLHLYTPPQQGLLPSVTGSRHDY*
>OoW9
MELRPPPKQLHHHRRRRGGDDDGEEEEEAGRLSLRAGGFWRRHDDEEKGGGRRGEIKEVDFFLASRDVAASR
RHEDGFRGTHGGGGDVNIGLDLLTTTTTGAAAGGVAAGAGEEDTAKNHRKEATTAVDAELRRVVEENRRLRG
MLDELTRSYSALYHQYLQVTQQQNYRHPDLIMNNNRSSLTQTHRTAAATMTATTQQFLEPRASSTAQQTAADA
ASDDEAGGGDASPSRSNTGGGNKIRRVGDQDGTAAPARENGEQAAAELPCRKPRVSVRARSEAPMISDGCQ
WRKYGQKMAKGNPCPRAYYRCTMAIGCPVRKQVQRCAEDKTVLITTYEGNHNHQLPPAATTMANTTSAAAA
MLLSGPTASRDGAAALLGHHHPAAMFHQSFPYASTMATLSASAPFPTITLDLTQTPAGSGAGTGAAGLLHALH
RPPAIHPGAAQAMPFAVPPQLAMYLPQQRAAPGMLPAVAAGMGARQPSVMETVTAALAADPNFTTALAAA
ISSVVAGGAHQALSTTPRTASGVAVAGDGNGNGSTAAAASPAPTAAEAPAASGSPPRLATRSCTTST*
>OoW10
MAASLGLCHETSYAYSYPASNTSSLCFPPLLADDIVGGGGGCSFGDFLELGHSEYSLPPPPPPPPQSVVVMAGG
GNEYGVSSSTMAATTSRIGFRTRSEVEVLDDGFKWRKYGKKAVKSSPNPRNYYRCSAAGCGVKKRVERDGDDP
RYVVTTYDGVHNHATPGAAAGHLPYPPSAAPAWSVPAASPPPAHAQAWGAPLHAAAAAHSSESSF*
>OoW11
MSSGGGGGGDRHGPYHQHGYLGRSDGGADYVYSSSDMESFFFSQPGGVGVGGGGGGAGAGGADEITPYSS
ITDYLQGFLDPSGLARHLDVACPSSQDTAVKQELSVDVTSHDSQGTGGAAGEGVAQATPNSSASFSSSDGEAEG
GKSRRCKKGQAKAEEDEKDEEDGENSKKPNKPKKKAEKRQRQPRVAFLTKSEVDHLEDGYRWRKYGQKAVKN
SPYPRSYYRCTTPQCGVKKRVERSYQDAAVVITTYEGQHTHHSPASLRGGGGGGGGHHHLFMPGVHGLPPSH
LMPAGFHPELMGLMHHPAMAAAAANPSMYFPSVAAAPPPAAAGGAISNDHPPLQQHHFTDYALLQDLFPST
MPSSNP*
>OoW12
MRGPLLQRAVVVMEHFNDWDLQAVVRSCSFPQPEPPRVGVGVSTAPEAAGAPVAPPARAPDQMARASALY
DLEYLDLDHKPFLLPGSSSSSSRAVALARGEDDGKSSHEVMISFPSAAAVSTSGAQPRSPSGRKPGIRTPRPKRSK
KSQLKKVVYEVPVADGGVSSDLWAWRKYGQKPIKGSPYPRGYYKCSSMKGCMARKMVERSPAKPGMLVVTY
MADHCHPVPTQLNALAGTTRHKSAPTDDDKPTSPGPAAGKAATGEGVVKCEDDIDGNELSAMAADGGAEDT
AAAAVDDDGELWPEGMGLELDEFLGPMDDDVFEFDHVLEDDGVLGRRLSL*
>OoW13
MAAGEEVMDRSTSAEDGYCSAGTDSPRAESVDEQGVAEESSPRGAQKRELPSPSASPSSPLPPAAKRSRRSVEK
RVVSVPIVECGDRPKGAGEGPPPSDSWAWRKYGQKPIKGSPYPRGYYRCSSSKGCPARKQVERSRADPTVLLVT
YSFEHNHPWPQPKSSSYHASKSSPRPTAPKPEPAADGQQPEPAENESSASAELEVPEMEPEPESEPVVKQQEEE
QKEEQKAVVEPAAVTTAAPAPPVEEEDENFDFGWIDQYHPTWHRSYAPLLPPEEWERELQGDDALFAGLGELP
ECAVVFGRRRELGLAATAPCS*
>OoW14

MDGEWSDGAVSSPTMSGGGGEQTKAREGVVIAAADCPGSPVSTSPAAAQRSAAGAAASPSGRPRRSAQKR
VVTVPLADVSRPRPKGVGEGNTPTDSWAWRKYGQKPIKGSPFPRAYYRCSSSKGCPARKQVERSRADPDMVIV
AYSFEHNHSATVPRAQNRQAAPQKPKAHACSPPEPVVEPEETHHGVTAGPVTGSGGAAAIEVRDEFRWLYDV
VSVTTSSTSPSDIDAADEMQLYDQPMFFGGAVVGTAALLPDEFGDVGGLGGEGLGEEEAMFEGLGELPECAM
VFRRRAGDGLAMGGGVKIEQPAESTAMT*

>OoW15

MEGSSSDGRYGGAGRCALVAELVQMQGMVRHLEAEMGPHGGGGGGGAAMAAGADERCRALVSDMLSSI
DRSISIARSCCAEAGRLMQPAGAAPESPPSADGSAGSDHGFDSRCRANAAGPCKKRKTLPKWSKQVRVSSVQ
DVGPLDDGFSWRKYGQKDILGAKYPRAYFRCTHRHTQGCHASKQVQRADGDPLLFDVVYNGDHTCPQGALS
AVVVDGQAAASAEQQQPPPLPPQPAEPPQEKNAVSVAFASMAVNASSSSPFVSPATSGCQVSYELGGGSMA
GVRNVPDVELASKTNSSIGDDMEFMFSLDADFLDNYKYYSSGYF*

>OoW16

MSGEYQFQDELAPLFARPGAGEMQMQQQLLPSSWFADYLQAGTPMQMDYDLMCRALELPVGEDVKREVG
VVDVVTAGGGGVGAAPLTPNTTSSMSTSSSEGVGGGGGGCGGGAGEEDSPARCKKEEDDENKEEGKGEEEE
GHKNKKGSAAKGGKAGKGEKRARQPRFAFMTKSEVDHLEDGYRWRKYGQKAVKNSPYPRSYYRCTTQKCPV
KKRVERSYQDTAVVITTYEGKHTHPIPATLRGSTHLLAAHAQAAAAAAAHQLHHHHHHHHGMAPPQLGSGAA
QFGRSGIGGVGGVDVLSSFLPRAAAAHHGMTPTTTTTTHGLTGGAISGGGAVSSATTSSAVTVAASPQSSSAA
ALQMQHFMAQDLGLLQDMLLPSFIHGTNQP*

>OoW19

MVELCGGEGEAQIMLASELAQLRAMARELEAKMDPDKVTARELCRALASSVDRSIRLAVSCFPPPEHSHRAAG
SPPPAAGNAGRDAAFKKRKGMAKVRRQVRVTSVQDTASLDDGLSWRKYGQKDILGAKYPRAYFRCTHRHTQ
GCNATKQVQRADGDPLLFDVVYLGDHTCGQAAAAQSAPPPLLPEHAGQEQQRHSSLLAVEAEGIHQQVVEP
MAAAPFLFTSTAAAGGVDDGYFSFISPANSDCQFSSDFSAGSVGVDMDHEARFEDLFLNTLEFFQSEIQNL*

>OoW21

MHASSETTTLGAAKGAGSGVGRGVRQRAVRVVVRARVESGELSMAMGSSASAAVMLELMTMGYQSAAYLG
ELLRAASPATAGEQQELAAEILRCCNRVIAKLSRGATGATTGKKRKAVESATSSSLPVTPTKRRARGAEAVREVRS
GTTADGFIWRKYGQKEINGCRHPRLYYRCAFRGQGCLATRRVQQSQSQSQDDPAAFVIAYYGEHTCGGDAAA
ACRDGELMPPAVINSGASNFTVAWNMGSHEPASLDVEQRSCDVDAPSETSQGWSPSFSSEVELDVVGFDLAG
ADEPWANSSASPVWEFLNGSFDWESVINSL*

>OoW23

MENLQLQGDDHANEAHAATALPHFPYFAVPPPPPLAVAPSASATSTDDHQHGPLEALEPPPCNHPDGLVDGP
QLATTTAATTAVPMMLPTMTSLDWQTLLQTCLPQVPPPVLEQQQAAADQYSGENDHGDLQATESSGAGNKE
KQVMAKGGAGRSSGKKKASRPRFAFQTRSDNDILDDGYRWRKYGQKAVKNSKHPRSYYRCTHHTCNVKKQV
QRLAKDRGIVVTTYEGVHNHPCEKLMEALSPILRQLQLLSQL*

>OoW24

MASSTGGLDHGFTFTPPPFITSFTELLSGGGVGGDMLAGTGGEERSPRGFSSRGAGRVGGGVPKFKSAQPPSL
PLSPPPVSPSSYFAIPPGLSPTELLDSPVLLSSSHILASPTTGAIPAQRYDWKASADLIASQQDDSRGDFSFHTNSD
AMAAQPASFPSFKEQEQQVVEASKNAAASSNKSGGGNGGNNKLEDGYNWRKYGQKQVKGSENPRSYYKCT
FPSCPTKKKVERSLDGQITAIVYKGTHNHAKPENTRWNSGSSATQVLQSDGDMSEHSFGGMSLENLLVSSDTA
ATPENSSASFGDDEIGVGSPRAGNGGSDVFDDDEPDSKRWRKDSDGEGISMAGNRTVREPRVVVQTMSDIDI
LDDGFRWRKYGQKVVKGNPNPRSYYKCTTMGCPVRKHVERASHDTRAVITTYEGKHNHDVPAARGSAALYRP
APPPSAATSSHQYMPNQPAMSYQSTGPQPYALRPDGFGGQGSFVGLGGGAVGGGSSLGGFSGFDDARGSY
MSQHHQQQRQNDAMHASRVGIATGLHPSGLATPSPSPLCKKFFIPILILAHGGGFFPVPVPMWVRGTRRVPT
PDNQTINMIICQQQ*

>OoW25
MAVDLMGFSPRGGCPSLETEQLAFQEAAAAGLRSLELLVSSLSAGEHHNHRPQHQSPPPLGEIADQAVSRFRK
VISILDRTGHARFRRGPVVGAAAAEAAAASPSSSPVSPLPPVTTQPAAAKSLTLDFTNPAKVAAASVTSTSFFSSV
TAGGDGSVSKGRSLVSSGKPPLAGGVKRKHPHPPCAAGDGHGAGAAHAHAHGGCHCSKKRKHRVRRTVRVP
AASARVADIPADEYSWRKYGQKPIKGSPYPRGYYRCSTVKGCPARKHVERAADDPATLVVTYEGDHRHSPSA*
>OoW26
MYMAAAAGASTPFNFRRHVPHADDVFSGSWMARRPSAPHGDGASFVAPTFGPAFRQHLDLLDYLSDDQGA
PPPPAAVPGASYVMPAPAMAPAEPVVPDSVAAAGYPRNVAAAVVAGEGRGRTTTDRIAFRTRSDDEILDDGYK
WRKYGKKSVKNSPNPRNYYRCSTEGCNVKKRVERDKNDPRYVVTMYEGIHNHVCPGTVYYAAQDAASGRFF
VAGMSHPDLN*
>OoW27
MNTFALFRDEVQKDSEKVHGHHAIDDEAGLLSLGLSLGSSAHAPLPGRRPLSSRGGQADAGNGGEVEAASAD
VYLDLPLRCDHAAGEPMVHPKRQRTTNGSSSSSICGEYGDGGAAVPAGRDDRSCIITAASTPANRPGRVVLRTR
CSAPTMKDGCQWRKYGQKTAKGNPWPRGYYRCTGAPGCPVKKQVQRCNHDTSVLITTYNGIHNHPITPYTA
ALPSSSSAAAMLTSSSMLSKLQRVMSRSIAMPTTQSSSSWSQKNYPIEADVVAKAIWDPKFQATVAAAVASYV
REQSIADEKGARELFNMAPPY*
>OoW28
MDSWIGQTSLSLDLNVGLPSTARRSAPAAPIKVLVEENFLSVKKDHEVEALEAELRRASEENKKLTEMLRAVVAK
YTELQGQVNDMMAAATAAANAGNHQSSTSEGGSVSPSRKRVRSVDSLDDVAHHHHHRKSSPPFVVTAAAAV
YASPDQMECTSAAAATAAKRIVREDCKPKVSKRFVHADPSDLSLVVKDGYQWRKYGQKVTKDNPCPRAYFRCS
FAPACPVKKKVQRSADDNTVLVATYEGEHNHGQPPQNDGGSKSSAAAAKQHQPPPSAAAAAVRQQQQQEQ
AAAGPSTEVARKNLAEQMAATLTRDPGFKAALVTALSGRILELSPTKN*
>OoW29
MAMVGAGDWPFAADEAYADSSAIFAELGWANGLAVVDAVGDELLPPLDPPGEATPPPLDLPETPAGSSADGA
ASSCSTDDGAAPDDADGGKPAAASTEAASKSPMAGKKGGKKRSRQPRFAFMTKSEIDHLEDGYRWRKYGQK
AVKNSPFPRSYYRCTNSKCTVKKRVERSSDDPSVVITTYEGQHCHTVTFPRAGAGAGSFSHIHTMAAAAALA
AGQMAAPAFSAHQQLYSNLQPPATMSLAAAATTPATSSSLLQLPLHCNQELQVVASSGYPSSSSSSSPASVPVD
KGLLDDMVPRAMRHG*
>OoW30
MDGTNNHGALMDDWMLPSPSPRTLMSSFLNEEFSSGPFSDIFGDNGSNKPHDGVGKSKAFVDSSREETTQL
AKKFESNLFGSNQKSISNGCLSERMAARTGFGVLKIDTSRVGYSAPIRSPVTIPPGVSPRELLESPVFLPNAIAQPS
PTTGKLPFFMHSNVKPSIPKKIEDETRHDRVFFFQPILGSKPPTFPVAEKGFSVNHQNQPSVTDSHQELSLQSSST
AAKDFTSATIVKPKTSDSMLDNDDHPSPANDQEENATNKNEEYSSDVIITPGEDGYNWRKYGQKQVKNSEHPR
SYYKCTFTNCAVKKKVEHSQDGQITEIVYKGSHNHPLPPSNCRPTVPFSHFNDLKDDHSEKFGSKSGQATATSRE
HATNGHLQDVGSEVLETKLSASLTTTEHAEKSVMDKKEAVDISSLLSNEEDDRVTHHAPLSLGFDANDDDAEHK
RRKMDVYAATSTSTNAIDIGAVASRAVREPRVVVQTTSEVDILDDGYRWRKYGQKVVKGNPNPRSYYKCTHPG
CSVRKHVERSSHDLKSVITTYEGKHNHEVPAARNSGHPSSGSAAAPQASNGLLHRRPEPAQGGSFAQFGDHA
AAAYGSAAGHRPADRFVAEAAGGGFSFGMLPRSMATPAPSPAITVPVQTAGRPPAMQGYPGLVLPRGEMKV
NLLPQSGGPVANGNAAAAAACQQLMGRLPKQHPRM*
>OoW31
DEGCRSAVRWWWVEMPLVRFEVRNEVGLGDPDLYGGGGGGGGNNWDLGAVVRFGCGGGRVSPAPTTTLL
GEAWEYDPFSSFLVPPMTTQQALPVWEGDDAAWMAPLPGLQTGGSCLDADDLCGVFFAAPAQQQPAVAAA
EVTPPSPTQAADDAPPPAHDNTQPATDQQASGGARAGGSRSARRKKKQTTKEVVRVAANGPAPDSWAWRK
YGQKPIKGSPYPRGYYRCSSNKSCAARKQVERCRVDPSFLLLTYTGAHTGHDVPLHRNSLAGTTRHKPPPLLPSA

AATAEA
>OoW32
MSSKKKRAAIDLSLKAERRRPEEGRGGDREASDDATAAEEDGDVNRGEGEPKEETGGEQEKVVEVVVDQGED
GSNEEIEYRTQQGEMMEEDKQPAAAANDDDGGDSDGAGASAEEKHMVTEATGGEGHDGGDSRTAMVQD
ELSEMQEEMERMKEENRMLRRVVDKTVRDYYELQMKLADYQQQPAADKPTETEVFLSLGATAAAGGGGGG
GFPEAKSKEQSAWRRRSVGSDDSDDGKEDLGLSLSLGASSSYDDDQKVEARPHDVDGVAAAMISGDGNKASR
GYALLESSKVQGAAPAAGEIAAAGGIASQSVNPANRKTRVSVRVRCQGPTMNDGCQWRKYGQKVAKGNPCP
RAYYRCTVAPGCPVRKQVQRCPEDRSILITTYEGTHNHPLPVGATAMASTTSAAATFMLLSSTTSSSSVSDASGG
VSTAPSSSYLSPYLLNSASHHHPSSSPLLMPGAMGGAGMQHLNLFGNSSSSSLLAPQAPGSNKYPWSPTHPPL
AGAGGKRPFWSTGGDGDKPAPAALPENVDAVMSDPNKFSAAIVAAINNFMGKDGESSSSKSSSKWGVVESLP
PHE*
>OoW34
MHTCMEGGGQLGTSLPNFYLLPDHHSMPLPPPLQLPCHPKLLQMPFDQEDQPGIHGVMLSSDHCGLYPLPAL
PLSNSTAAAATVALGKHSAAAGSMPNISGVEEVATSVTKAGNESITCNGSTTWWRGSTMAAAGEKGKMKIRR
KMREPRFCFQTRSEVDVLDDGYKWRKYGQKVVKNSLHPRSYFRCTHSNCRVKKRVERLSTDCRMVITTYEGRH
THSPCDDNSSGEHTNCFSSF*
>OoW36
MYACMEGSQLEIACLPAAALYAPLCPYTPPSPPSLLAPLPSLQHKLPQLPPLVHEHAAGTNHGVMFSSDHGCLYP
LLPGIPFCHDSASAACDKPAGFAPSAEAAGTSTAAKVDSEIAAAATATTCHGPNSWWKGSEKGKMKVRRKMRE
PRFCFQTRSDVDVLDDGYKWRKYGQKVVKNSLHPRSYYRCTHNNCRVKKRVERLSTDCRMVITTYEGRHTHIP
CSDDTAAAAAGDHTATCTFTSF*
>OoW37
GGRPSGEVVPSDLWAWRKYGQKPIKGSPYPRGYYRCSSSKGCSARKQVERSRTDPNMLVITYTSEHNHPWPT
QRNALAGSTRSHHSKNSGGGSGSKGSQNEKSQQQPSVKEEQKDQATTATTTSTITTTNSASPVVVKEEEALA
GSSEALELERVMDTAAGVVDHSELMDHVFSESYKPMIPETGQPDDFFADLAELESDPMSLIFSKEYMEAKPSG
GDHAQEKAIAKELDPFDMLDWSTTNSSAGSSFEQGKRG*
>OoW39
MEEELCGNNWDLDAVVRLGCCRRRISPAASASSAAAQQVDPFASFLLPVTQPTTVAMEVAEKELGVDAGWSF
PELTVRDGGGLGRNADELLKAFCAAFPSSSSSSSSLPTPPPPPPQPEQQKPVAVVVQENLPAPTTAAPASAPARA
QPAAVRQVPGGVPRSKRRKNQQKKVVRHVPADGVSADVWAWRKYGQKPIKGSPYPRGYYRCSSSKGCPARK
QVERSRSDPNTFILTYTGEHNHSAPTHRNSLAGTTRNKLPSSTSASAQPQPPPPSVVVAGGAEAASHQSSSPSG
LSPTTPLRTPSMEEDEEDDDELLVEDMEMAGEDELLFLNGDDATAAALDATPMSSLFDIDEPFLPSPWTEPAAA
GS*
>OoW40
MKNSSNKRSLVADQWHPSSVCCDHRAALREIARGQSLVTQLRAIVLPALQSDERCDLAAQMLEGILDCSRKAIS
ELQLLLSSSDASARDDDDLDDKRRVRKIISSHDDDHCSSKAAEDHNAKPLRQHKRRRFGDSVSLETPVPHYDGH
QWRKYGQKHINNAKHPRSYYRCTYRQEQKCKATKTVQQREDLHHANSYNGDHPIMYTVVYYGQHTCCKGPA
ASAEDHAVVEASQISTESHCQSPSSSSDLQPKAHAGDSSQCSNISVTCSSSVVVEDCNKLLDMLPPADELTTDVL
LFDMTAYTPLDLDINWEMDAYAQALWV*
>OoW42
MADPLPAVARGGGVRDGGEQGGTAGQLVVTPSRLRTAVASMLNRTGHARFRRAPVVVQEEEEEAAAAAAAR
DVIVRCDGLSASASSSFPSSVTGVTGDGSVSNARALLRAAGGRSAGCDRPPPMQCASDYASGRLKRNSDDDGE
RCHCSKKKRKASWRARRRIRVPAISSRNADIPADDYSWRKYGQKPIKGSPFPRGYYKCSTLRGCPARKHVERDP
GEPAMLIVTYDGDHRHGEPTDRLEDALASTV*

>OoW45
MTSSMAPATPPYAQVMEDMEKGRELAAQLLGFLRDSPEAGRIVDQILHTFSRAMQALDKAVSAAGGEGSEVQ
SGVTCGGGASAGGKRKASAGGAAADRRANCRRRTQQSAGKTVVVKNLDDGQAWRKYGQKEIQNSKHPKAY
FRCTHKYDQLCTAQRQVQRCDDDPSSYRVTYIGEHTCRDPATAPIAAHVIHQVAAGDDDDGGLHAGSRLISFVA
APAPVEAAAAPTTSTTTTVTAPGPLLQPLKVEGGVGSSDQEEVLSSLTPGNSAARGGGVGAGPFGPDQGDVTS
SLHWTYDAVAGAGMEFFRDDEGVFDLDDLWV*
>OoW46
MALDSVVPSYPSDLGSSRPTSTRTPQQQRVSPRKEERTWTTDTYAPYDDGHQWRKYGEKKLSNSNFPRFYYRC
TYKNDMKCPATKQVQQKDTNDPPLFSVTYFNHHTCNSSSKIVGSTPDSTGQSSSRKAISICFNSHGTTGEQPTFL
SSSASLLSPSIQSYRSNQQPDMNTYSRQFQWTDTSSSTSNAPVKTEADDYAEVSASPNTTGALSRTLLPIGQSRC
IEYFHFL*
>OoW47
MASPGGGVGDGSGLPAAEPHEVIDDLLEARKEAVMLHNMLQGTPSSCVVASTRQLNQLIDGVVSRLQSSSLS
VMSPGGGGRRGSGGRKKKSATPVAGPHRRSSSGRRRSKSPLVKTVTTKEMEDGKQWRKYGQKHIQDSPNNP
RSYYRCTHRPDQGCMATKQVQTSESNQSEFVISYYGEHTCRDPSTIPFVVEAEAPAADYANLISFGPGAIASTSR
DPLQGRRSFINAEAVDPTPSCSFANCHSHSPLSPECASEAAALSSSLPLSAVVGSAVTTPSTSIVGSAPAEYDWPS
GIAGGDMAGSFPSSPSSLGFMTGSLGSFGNLPGDDDDMFDFDP*
>OoW49
MSGASHDHLQLGGAGAGEGFPFHDELASLFAERPPNAAMPGMQQPPWSFIDYHHLMQESAPTTPPLDYEA
FAGEFDDDVAAAAAGGPPLEEVKRELVVDGVGLLPVGGGGAAAAAAGPMTPNSMSVSSTSSGAGGDDEFAG
KCKKEEGDGDDDGKEGSATTKGDGDGEDKNKKGGGKGKGKGEKRPRQPRFAFMTKSEVDHLEDGYRWRKY
GQKAVKNSPYPRSYYRCTTQKCPVKKRVERSYQDTAVVITTYEGKHTHPIPATLRGTAHLLGAAHHHGGLQYHP
GHFAAGHRLPPPPGAASAIDALGGLLAPPQHLHAMQHQMQLAAASGGGATGSVHAAMQQMQPDHAGLA
AIIASNTGVATTTAPGSGTTTPLRMQHFMAQDYGLLQDMFIPSSFMHNDDANNHHR*
>OoW51
MITMDLMSGYGLVDEQVAIQEATAAGLRGMEHLILQLSQTGTSERSPAPAPAQEQKQVDCREITDMTVSKFKK
VISMLNRTGHARFRRGPVVAQSSGPAASEPAPLRSSPSAVSRPMTLDFTKAVSGYGKDAGFSVSGLSAASSSFLS
SVTGDGSVSNGRGGGSSSLMLPPPPATSCGKPPLSSAAAMSAGAGHKRKCHDHAHSENVAGGKYGSTGGRC
HCSKRRKHRVKRTIRVPAISSKVADIPADEFSWRKYGQKPIKGSPFPRGYYKCSTLRGCPARKHVERDPADPSMLI
VTYEGEHRHTPAAGQDPPPPPPAPSPPLALPLA*
>OoW52
MAVTDSVCLSDEQEAVAVTEVAQVYELIKTQQPLLLVHQQPQQLAHGLLSHALRALNVALSVMNQPHHQHAS
SSAAAAAVPVMSMIKAEAATPANSSSPAADVADNHVVGKPRRSSPAKRRRINCEDKSSWVNHTVVPHEDGYQ
WRKYGEKNIQGTHFTRSYFRCTYRDDMGCQATKQVQQKDNNDPPNFQVTYSNDHTCNFVRTTTRIINNTNN
NPAAALHSLTANPNDPDDDDDDDTIFTKMIKQEKPAAWLPPPDLTAISNNSDETPVLHVCQEVAPCSSNSSVIS
HYADEFDQHQMLETTVMEEALGLGADLDDPYFYDPSLLVLYENLMNCY*
>OoW53
MASSTGGLDHGFTFTPPPFITSFTELLSGGGVGGDMLAGTGGEERSPRGFSSRGAGRVGGGVPKFKSAQPPSL
PLSPPPVSPSSYFAIPPGLSPTELLDSPVLLSSSHILASPTTGAIPAQRYDWKASADLIASQQDDSRGDFSFHTNSD
AMAAQPASFPSFKEQEQQVVEASKNAAASSNKSGGGNGGNNKLEDGYNWRKYGQKQVKGSENPRSYYKCT
YNGCSMKKKVERSLADGRITQIVYKGAHNHPKPLSTRRNASSGCAAAACADDLAALGGAGADQYSAATTENS
SVTFGDDEADNGSHRSEGDEPEAKRWKEDADNEGSSGGMGGGAGGKPVREPRLVVQTLSDIDILDDGFRWR
KYGQKVVKGNPNPRSYYKCTTMGCPVRKHVERASHDTRAVITTYEGKHNHDVPGRGGGRAPAAPLPAPSASG
AIRPSAVAAQQGPYTLEMLPNPTGLYGGYGAGAGAAFQRTKDEPRDDLFVESLLC*

>OoW54
MARTMASLAAIVGKPTAASAQLVAEGRESAARLHALLSRCHQLLVGSSALHGPVGLAEQILLCFDRALAKLHVA
GAEDDDAAGNGRKRKPGRVLAAASAAASSKRMRASNGGGNGARIERKATMDDKFLWRKYGQKEIKNNKYP
RFYYRCSYKDDHGCTATKQVQQSEDDDTASPVYVITYFGEHTCCPGIDAAAVVVDGGEEKELSRAQMVISFASS
GGDASVCWPSSGDDAQNNSETSQESCTAADVSDEPILLKPTPPAPELPDLKPMDGCLLDGESLFGMDELIYFHE
LSAALGLLDRDWGAPV*
>OoW55
MSPVPSPHQSHHLGHGSRKEKRMRKVDTFAPHNDGHQWRKYGEKKINNCNFPRYYYRCTYKDNMNCPATK
QIQQKDYSDPPLYSVTYYNEHTCNSAFLPLSPSEFQLQTASGKAVSICFESSGAQEPMTNASSPSSSAARRSTPSE
NKNQPLPRHSEAYSWGVGVVEQKPACTELQSCSTECQDAFSAGTIPEETVDAGRFGSIRFFHFL*
>OoW56
MGDVLRAQATAAADEVCVWPGELDEHLIGELLVDDGLFVPAAEHPDLYYSFDAGSSAAAAAAAPCNGGGSAE
HEPPRPAPAVSRALCSVYSGPTIRDIEKALLSSSSRSPYPSGHRYSSLYFRRVEAESKYTSKVRSCGGKMPADGYK
WRKYGQKSIKNNRHPRSYYKCTSSRCSAKKHVEKSTDDPEMLIVTYEGSHHHGPQPLFPPHIAQPPPTTSVVDF
SAAGAEPPSSSAAARKRKNDVRPGFSPTTSEDDVDGAGLRPGWPQDDETSRDDAELQRRGAAPRRVATDHS
CDDGGGSTSASSVARADAVTALSSDSPPTIWSCLDWPWSQETLFL*
>OoW57
EEERVLSHGDVVLLRCDLTILRGPHFLNDRIIAFYLAHIHHDDDDDLLLLPPSIPYLLSNLPDPASVAAVAEPLRLAS
RRLVLLPVNDNPDVSHADGGSHWTLLVLDNSVPRFVHHDSLPGTNLPPARRLAAVLRPLLPATPIPLIEGPAPRQ
TNGYDCGVFVLAVARAICHWWPTRDRTNSDWLEAVKREVNADSVKAMRTEVLQLIQTLIQDKKNNTNQNKN
NGPSTQLPCCPSIASTSLCLSQHTRLWWDMQSHKKVAAVKPVASRPSSRLRSFSMLQKDSTATDSPWLTSLEEE
IILRRPKATRFTPSLGDSSTEIAATRLEDNDTHTTYDQKKADTGQVASWDNLTVSQSVRKPNVRVKNSLSYDGYS
WRKYGQKQVKGSEFPRSYYKCTYPTCPVKRKVEMTPDGRIAEIVYNGEHNHPKPHPPRKPSLSASVETLVTTND
AGLENHQAIGSDAVVEAFKGVCHCLDGFRNGNEISDCKKRYAYPVIFIQNLLLFLCAKNVQVVA*
>OoW58
MDSLQPAGDQLGLLLPQLPAYLASSSSAPPASMAALSPAGDDWAASLLLPAGGSAAAGVGEDDLSGGVMAAA
AAAAAESSCGGSSTVTSSGVTEAATTTGGMTTTRRVRGNGKKAGGGGRTPRFAFHTRSENDILDDGYRWRKY
GQKAVKNSKHPRSYYRCTHHTCNVKKQVQRLAKDRGIVVTTYEGVHNHPCEKLMEALSPILRQLQLLSQL*
>OoW60
MASNSQPTTTGAGRGRGQGDDEEQTPPPAPPPDTASAVGGGDGVQLVMPEDGYEWKKYGQKFIKNIQKNR
SYFRCRDQRCGAKKKVEWHPHDPTLNLRVVYDGAHHHGSPSSAAGEEAAGAANQYDLSTQYFGGAGPRSQ*
>OoW61
FMMHPGRPSISIQCVDWTFDGKDDDNVGTQEEEHRTLQRQHHTVKEGSNEKSPVKQKDPREGAHKSVDGR
KTLRRIWVKVSSTVDDGFSWVKYGQKEILGAMYPRSYFRCTHRQTKGCLAMKQVQPTDEDQLILDVIYYGEHS
CDQSACSGNSQMRSSRPAASSNLQEPQQPGLEQSRPAAKWRRKTKMERKTQVRVSFLQDVGPLDDGYSWN
KYGQKDILGAKYPRAYFRCTHRNTQGCVATKQMQRRDADPLLFDVVYHGAHTCSQRASLNEQASTEQSQSSS
TITYTAGSVEDDDEEGVTSATNFLPMDDMLDLGGGDVTDMDFPSFDFDAMEALLG*
>OoW62
MDDDDGSSSPTDDSAAAGLLPLFSRSPVEDLEEKLRRATEVNARLTRALDAILGGHHAHQRAVLAPSPSPPSAT
TRARSVSTSCAAREDAPAVAAAAASTAGPSRQPPPTAEPRPKVRTVRVRADATDTDANNMAETVKDGYQWRK
YGQKVTRDNPYPRAYFRCAFAPSCPVKKKVQRCAEDRSMLVATYEGEHNHALSTQTTEFVGSGCTSQHAGGSS
SLPCSISINSSGRTITLDLTNQGSGSIASCGVQAVSGELVTVLSPEFRRHLVEEVVQVLKNDAEFVEAVTNALAARV
VDQIPHSPVDL*
>OoW63

WMKFEAGAMPNLRKLKLRFDVRRADQHDTIPIGIEHLSGLEEISAKITVADYLCRRFAESALTNAFRMHPGRPS
VNIRCVDWTFDGKDDSNAGTREEECRTLQKQQNIVKESSTEKCAVLEKDPGRGANKSVESREKPHMVGRHLK
SNTEDDGLSWIKYDQKDILGAKFPRAYFRCTHWSSTKKKGCMATKEVQRDDDDPLLFDIVYHGDHTCAQTNN
EQPVRSLTRSTDEQSQQSGLELSSPYDWHGHKLSAKAQVTKMTTQVQVRLSSVQDVEPLWDGHGWRKYGQ
KDIVGAKFPRFYFRCTHRNTQGCVATKQMQRRDADPLLFDVTYHGKHTCAVEAAHSSDKLSGRLEQSSTVTSV
KTTDSTDGEDVASTANFLTEPDMLIPSPGLNDLFWDIEDIWRYQP*

>OoW64

MKNSSNKRSLVADQWHPSSVCCDHRAALREIARGQSLVTQLRAIVLPALQSDERCDLAAQMLEGILDCSRKAIS
ELQLLLSSSDASARDDDDLDDKRRVRKIISSHDDDHCSSKAAEDHNAKPLRQHKRRRFGDSVSLETPVPHYDGH
QWRKYGQKHINNAKHPRSFYRCTYRQEQKCKATKTVQQREDLHHANSYNGDHPIMYTVVYYGQHTCCKGPA
SAEDHVVVEASQISTESHCQSPSSSDLQPAAHAGDSSQCSNISVTCSSSVVVEDCNKLLDMLPPADELTTDVLL
FDMTAYTPLDLDINWEMDAYAQALWV*

>OoW65

MEAYNYCMMVGRERELVAELRHLLFPSPTPTTPAHRATSTLAGDQCLPPGLSGGGRRRGRKRVRRGNDNVKL
LQADDDHDDQETVVADHGDANAKPLPNFTKTSRRKQQTTTSTMVTTVPDFDGYQWRKYGQKQIEGAVYPRS
YYRCTNSTNQGCLAKKTVQRNDDDINGGGGGAAPYTVAYISEHTCKSIESSLAPVILDTTVRTNNHHPPAAAPV
VTAAAAESAAQSPATSSSSDMIMTSSTRSSTSGTSSGETSWSGQHGAYACRQLIAADEDCWDDTPPTTITTSDG
GNSCAEEIDLLSGPIRSPVHIAADGNWMDDLLLVSDGLIDISNANISHLFCF*

>OoW66

WRKYGQKPIKGSPYPRGYYRCSSSKGCSARKQVERSRTDPNMLVITYTSEHNHPWPTQRNALAGSTRSHHAKN
SSSSSSSASSASKNNSSHSGYHHQKPLVKAEPNDQAAAATKTTSTVPVKEEATMVGSSEALAKQKSMEDAAAS
ATAAAVEHSDLMQQMFSQSYRPMIPEAGGHHDDFFAG

>OoW67

MAASIGLNPEAFFSNSCSYSSPFTASYTPEFSATTIDADFSGELDFDYSLPAPAFARAGEDYPDNENTMMRCESE
EKMRARVNGRIGFRTRSEVEILDDGFKWRKYGKKAVKNSPNPRNYYRCSTEGCNVKKRVERDREDHRYVITTY
DGVHNHACPAAAAAALQYGGAGDYYSPPLSSAGSPPAAYSAGGSLLF*

>OoW68

MAVDLMGCYAPRRADDQLAIQEAATAGLRSLEMLVSSLSSSQAAGAHKASPQQPFGEIADQAVSKFRKVISIL
DRTGHARFRRGPVESSPPAAPVGAPPPPPPPPPVAAVAPLAPTSQPQTLTLDFTKPNLAMSAATSVTSTSFFSSV
TAGEGSVSKGRSLLSSGKPPLSGHKRKPCAGGHSEATANGSRCHCSKRRKNRVKRTIRVPAISSKIADIPPDEYSW
RKYGQKPIKGSPYPRGYYKCSTVRGCPARKHVERATDDPAMLVVTYEGEHRHTPGPLPAPGAAAVAAMPVSAA
VATVSAGNGHV*

>OoW69

MEGVEVNGGEGGGGWSAQLVVAELVRVQELLRQLEVHLNAPCSVELCRGLAAQIIALTDRSIGIASRSFSGAGG
VAGAHFADTPPPALTSCTPSPLSDGSDHQPFRTTNPKKRKTTARWTSQVRVSAAGGAEGPADDGHSWRKYGQ
KDILGAKHPRGYYRCTHRNTQGCTATKQVQRTDDDASLFDVVYHGDHTCRPAATATAAKRPHAQTLLQSLSASL
TVNTDTNTLLAATAAAVTPLTPDNRATAPPQQQSVSPSLASPVASDSYGLAAGYRDWHCCDGDLQEVVSALAT
VTSAPEHAAMDAGDFMSYCFDFDPAVYGSIGTPSFFP*

>OoW70

MTAAPGSLPLVNSRPVALSLAASRSSFSSLLSSGSSLNLMTPPSSLPPAAASSPSSYFGGVSSSGFLDSPILTPSLFP
SPTTTGALFSWMTTATATETTPESQVQGGVKDEQQQYSDFTFLPTASTAPATMTGVTATTSNSFMQDSMLMA
PLGADPYNGEQQQPWSYQEPTMDADTRPAEFTLSGVTAAGGDVAGNGGYNQVAAPAAVGFRQQSRRSSDD
GYNWRKYGQKQVKGSENPRSYYKCTFPSCPTKKKVERSLDGQITEIVYKGTHNHAKPQNTRRNPGSSAAQVLQ
SGGDMSEHSFGGTAATPENSSASFGDDEIGVGSPRAGNGGSDVFDDDEPDSKRWRKDSDGEGISMAGNRTV

REPRVVVQTMSDIDILDDGFRWRKYGQKVVKGNPNPRSYYKCTTMGCPVRKHVERASHDTRAVITTYEGKHN
HDVPVGRGGGRAPAAPLPAPSASGAIRPSAVAAQQGPYTLEMLPNPTGLYGGYGAGAGAAFQRTKDEPRDDL
FVESLLC*
>OoW71
MDPWISSQPSLSLDLRVGLPQAAAAVAMVKPKVLVEEDFFQQQPLKKDPEVAALEAELQRMGAENRQLSEM
LAAVAAKYEALQSQFSDMVTASANNGGGCGGGGNNPSSTSEGGSVSPSRKRKSESLDDSPPTPPPPPHPHAA
AHHMHVMPGAAAFADQTECTSGEPCKRIREECKPKISKLYVHADPSDLSLVVKDGYQWRKYGQKVTKDNPCP
RAYFRCSFAPACPVKKKVQRSAEDNTILVATYEGEHNHGQPTPPPQSAAQNSDGSGKSAGKPPHAPAAPAPVV
PHRQHEPAAVIINGEQAAAASELIRRNLAEQMAMTLTRDPSFKAALVTALSGRILELSPTKD*
>OoW72
MENFPILFATQPSSSTSSSYHFMSSSSGSHDHRHHHGLQAGGNGGGGSLSHGLFMGSSSSSRMEELSNSKDD
VDGATRSPHGGGGHGESSAGDGAGEGDVQAAAGGGRKKGEKKERRPRFAFQTRSQVDILDDGFRWRKYGQ
KAVKNNKFPRSYYRCTHQGCNVKKQVQRLSRDETVVVTTYEGTHTHPIEKSNDNFEHILTQMHIYSGLTPSSAA
HASSSSPLFPSAAAAAASHMFQ*
>OoW73
MKEDMIKGDSKQLASLYMKEEMIKGERQLGTHEDRLKDEVIKDADKISDGNLFKSLQNIPSTKEEGQDDKLAST
RAEMGEVREENERLKTLLSRISHDYRSLQTHFYDILQQGRAKKLPDSPATDIEEREFVSLRLGTSTSKCKKEDKSTT
STEVKGSIEDFLKIKGGLSLGLSDCTVDANNREKVQPDVMTLSPEGSFENARDDTTETTEQWPPSKMLKNLRSV
GTEAEDDIAPQPQVKKARVSVRARCDAPTMNDGCQWRKYGQKIAKGNPCPRAYYRCTVAAGCPVRKQVQRC
ADDMSILITTYEGTHNHPLSVSATAMASTTSAAASMLISGSSSTSLASYPAAASPALTFDASKPPVVGGRPFFLPT
AAASITSTPSYPTITLDLTSPAAATSSHAGFSLSNRFSSSFTSSHAAARYPSTGFTFSGSGPSSTPWPGYLSYGASLA
AHPYNAGGGKSSSSFEAALSSINGSRQHGGGSAALYQMQQKAAPGAAPPPSVITDTIAKAITADPSFHTALAAA
ITSYVGKKGSSAATGGEDSKVGLKWGEHLGLGLAHSSPSTATTAAAAAASSSSQMFLQPSLGLSGSTTSASTSSV
ANREQAH*
>OoW74
MQSMESNGGGRLVVTELSHIKELVKQLEGHLGGSGSPDLCKHLASQIFSVTERSIGMIRSGHFGGHRKRAAAG
DLDSATPSPLSDVSDLPFKATKKRKTSTEKKRHQIRVSSTGGVENPPVDDGHSWRKYGQKEILGAKHPRRGYYR
CTHRNSQGCVATKQVQRTDDDATLFDVIYHGEHTCVQKAVAAGAGKPQPESDTNAAQSRLHDLSSGLTVKIEG
LTATEQPPPQQQQQGVSWNAMPPFCLSSPVSGLAPPEHNPFSAPSTPENCLGVDGMSSASPATSDSNHLFVA
APLHQVAGDVAWRDTEIQEVVSALVAATTTAQPATAAMDADFSLDGFEFDPGFTIDITSFFA*
>OoW76
MDAAWRGGVGCSPVCLDLCVGLSPVREPLAARHELLDRPAGCRGGGDSRAMNNDEAKILEAKVTQMSEENR
RLTEMIARLYGSQIARLGLDGWASPPRPVSPQLAGRKRSRESMETANSCDANSNRHQSAATADADHAESFAAD
DGTCRRIKVSRVCRRIDPSDTSLVVKDGYQWRKYGQKVTRDNPSPRAYFRCAFAPSCPVKKKVQRSAEDSSLLVA
TYEGEHNHPHPSPRAGELPAAAAGAGASLPCSISINSSGPTITLDLTKNGGAVQVVEAADGAPPPPDLKEVCRE
VASPEFRSALVEQMASALTSDPKFTGALAAAILQKLPEF*
>OoW77
MSPFYSSLLSRSPAEYQLVGAGYAGEDDGDDDDMAAVDAVSSYLSFDMDVVGEEYYTPEVDFHSKQNTSPPPV
AAASLEAGGSADEVSREQSRREAVVLNSPGLAVNHGKMDKGPAPASGGAGPGLPRSKAGSKIAFKTRSDVDVL
DDGYRWRKYGKKMVKNSPNPRNYYRCSSEGCRVKKRVERARDDARFVVTTYDGVHNHPAPLHPRPQLPGSG
GYSIAGAPAVGAHRLGLEEAEVIALFRSTAATSLQLP*
>OoW79
MAFSSEGGVPAERVAAAANDLVEVREGLVRLRGFLPPPQAEQSSSTSPCAELMDAMMSKLMSAMAALGGSG
DVAGEVDAAGRWTSVAGSADPMVVRQAGESSAGRTRKRGGGGSRSGRGRSSNKRVAATLDDGHVWRKYG

QKDIQNSPYPRSYYRCTHKLDQGCAATRQTQRCEVDPSNYDITYYGEHTCRDPSTIIPTAIANAGAASDGPNNII
SFATGGVASRLAREGTTTSAATQLSSSWGTSGGGDDVFSSSGERFMQWDELAAAVVHVGSAGVTSSTVGSAP
AENDGGNGDTAAGGGGDGGGGGGGAGSFPSSPSAGSLGFVVGPLGSIGDVDDFFPFDP*

>OoW80

MDMMEEEAANAATAQAAAGDLADVVACANARAFLVSTPHRHHSPLHPLPLPMPPPPHQYYPAAQITIPYHQ
HGAAPDLRRPTTIAYADTAAAVPPSTVVDSYHLTPAGGGYGMPRPLAVQISQHALCSGGDVVMGGGSGAADD
GEDAIRISPLTPSAHHQMMKRKNNEVKKVVCIPAPPATSGRGGGGEVIPSDLWAWRKYGQKPIKGSPYPRGYY
RCSSSKGCMARKQVERSRSDPNMLVITYTAEHNHPWPMQRNLLAGYARSHHTHAIASTSRHKQQQQQTICN
NQLPALITSSSSSSSSPCNLYADVLGGQQANMMMTTTEAGTGVPGIQHSAADEVFAELEELEPDNPTMINAN
MAYSMTTTTTSRPGVSSYDHQWHKF*

>OoW81

MAAHEGGGNGARRPPALPPLPTLSLPPRSAAGTLFSAESSPGPLTLTAALFPDAPSPAFQGSFTQLLVGAMGSPA
ASAPAPPSPFPVPHGLSPTAFLGGSPGLFSPTGNFEMSHQQALAHVTAQAVRSPYSMINQSDYSLPFSSTTTSVL
ASRHVNSSANVSSPREIPTLPSHTGNSNIESTEVSHGFQTTALTEDKPADDGYNWRKYGQKAVKGGEYPRSYYK
CTHLSCPVKKKVERSSDGQITQIIYRGQHNHQRPPKRRFKNGGALLNEADVSPEKEDASTRSEQGSQDYSGKFK
ASNDGGPLSSRRGDGGEQISGSSDSDDQGEEEVKVEGGATSDGNANKRHVPPPAQRIIVQTTSEVDLLDDGYR
WRKYGQKVVKGNPHPRSYYKCTYQGCDVKKHIERSSQDSKAVITTYEGKHSHDVPAARNSSHSSANANISASS
NLPHRDRRQRSSRRDGLRNASSVSSLQLKEESG*

>OoW82

MKNKSRCRYLPHSSAPCSTDDTRCFRCDHRPAIDEIVREQSLVTQLRAVVLPALETKADGRAEIVAQLFGSILDCS
RKVISALNSRYVGESPPDDEIVDKRRVKRKNSEGKKGDEVKVKSHEHKRRRYTNSTSQVTPVPHYDGHQWRKY
GQKNINNSNHQRCYYRCTYKHEQNCKATKTVQQLDSAGETIMYTVVYYGQHTCKTNMSNAPLHVVETSTPQ
SISTTCSDDLGDYSQKIENMHTPELAEVCSDELDSYHAIIGAEHSALGLEDEHMHRLLDTFACGAVDLDSWEIDA
IVRSGFC*

>OoW84

MATRLPKSERSPSLPQPPGDQRDAAIQELRKGSELATQLMAQLELIPERELDRRDDALANVRNLSMSLSSSLYAL
RCEREHYCSSSSSGAGPAAVTSVSGGGGERKTKRRRGKHGEELIETVFITTTPENDGFHWRKYGEKNILNNEFR
RLYYRCGYSDDRKCQAKKYVQQENNKQPPEFRVTLTHEHTCNTLFPDQQASSSSTNSQVLDFTKASMSSTMDS
HGGAPVLKEEEDEIPSIDESTRSIMSTIMPNYYDYDDGAGWQ*

>OoW87

MADSPNPSSGDHPAGVGGSPEKQPPVDRRVAALAAAGAGARYKAMSPARLPISREPCLTIPAGFSPSALLESPV
LLTNFKVEPSPTTGTLSMAAIMSKSANPDILPSHRDKSSGSTHEDGGSQDFEFKPHLNSSSQSAAPAINDPKKHE
SSMKNESLNTAPSSDDIMIDNIPLCSRESTLAVNVSSAPSQLVGMVGLTDSAPAEVDTSELHQMNSSGNAMQE
SQPESVAEKSAEDGYNWRKYGQKHVKGSENPRSYYKCTHPNCEVKKLLERSLDGQITEVVYKGHHKHPKPQPN
RRLSAGAVPPSQGEERYDGVATTDDKSSNVLSNLGNAVHTAGMIEPVPGSASDDDNDAGGGRPYPGDDAVE
DDDLESKRRKMESAAIDAALMGKPNREPRVVVQTVSEVDILDDGYRWRKYGQKVVKGNPNPRSYYKCTNTGC
PVRKHVERASHDPKSVITTYEGKHNHEVPASRNASHEMSTPPMKPVVHPINRNMPGLGGMMRACEPRTFP
NQYSQAAESDTISLDLGVGISPNHSDATNQLQSSVPDQMQYQMQPMGSVYSNMGLPAMAMPTMAGNAA
SSIYGSREEKPSEGFTFKATPMDHSANLCYSTAGNLVMGP*

>OoW88

MAAARRVEGGGSLWGPPPPPSSGGGPPQLPAAAVEGLLDAPFPSGGGGGDGWPPLSGTAVLLGYPQGNFQI
FPEQDLVPLTAQDVHSKCITLGRAENPSFIPLATSALVSQHTRSSSVNVTPLQEILTLPSQISNVNTESIGVLQGLPA
SSIVLDRPADDGYNWRKYGQKAVKGGEYPKSYYKCTHPNCLVRKNVEHSADGRIIQIIYRGQHTHERPTKRRFK
DCGGISDDLDDFSGTTGISVRSQTDYDDYCRKPIIPSGTMVGPLVKKIEDGDDQLSGSSDNQDEHDDEVRTADG

ASGDASSNERNVPAPGQKIIVSTTSEVDLLDDGYRWRKYGQKVVKGNPYPRSYYKCTYLGCDVKKQVERSVEEP
NAVITTYEGKHTHDVPAARNKGHDVANASLLQNTKSNTYSTEQAYTTITC*
>OoW89
MVAVPCFLASPAFAGHFSMSHQAALASVTAQAQIQLQSPTTPYSEGLPSPFPMTPKAVMPLQQAPSGTEGSV
RRPVLEKSASFQSRPHHHVSVNMVGDGFNWRKYGQKQVKSSENSRSYYRCTNSNCLAKKKVEHCPDGRVVEI
IYRGTHNHEPPQKTRFVKERVAHITGSSGDDETLRLVNTEIIESRSPGCKLEPGAVSEASEQQLFCSSDCEGDAGN
KSEDDHPSTEPQPKRSRIIETSTPLTPVLRTVREQKIIVQAGKTSDGYRWRKYGQKIVKGNPNPRSYYRCTHDGC
PVRKHVEKAPDDDNNIVVTYEGKHNHDQPFRSNSESKDGPVPMIIPAETISEQPSTMTSTLDQKQPISMLKGG
DNEPTKDKTSEIGGEKAVEPAQPLISIKTNPDDMKNTLLKDTSAVVPVQNN*
>OoW90
MAGSSDHGSLMEDWMPPPTPSPRTLMSSFLNEDFVSGSFSNIFNDHESNKHQDQFERSRELVDLSKEVPSQS
ARPAFQRYASLDHSMVSPTQRSNSHGGLAERRAARAGFSVPKIDTSRGGSSAVIRSPVAIPPGLSPTTLLESPVFL
YNAMAQPSPTTGTLPFLVASNAKSTIPSATKMDEDHTFGNDNFSFQPHVGSRLPNFSAAEKGLSACHQNQSLS
NIHQQESSLQSSFTAVKDTTDEKNVKTKTSDSMFGDNHSSSDEQEGDETKQNGEYSMPPPNHRSGVPLSHTN
NPEVTVLENHGSQTGHNSASLWDNGKNDRLQDAQSEVVETRTAACLPISTNCDTSIIESQDAVDVSSTLSNEED
DRATHGTASIECNGDGDETDSKRRKLDALTAATAAITTTSNIDMGAAASRGVREPRVVVQTTSEVDILDDGYRW
RKYGQKVVKGNPNPRSYYKCTHQGCSVRKHVERASHDLKSVITTYEGKHNHEVPAARNSGHGSSGSGSVPSAP
QANGSQRRQEPVQASFGQFGGTAPFSSFVLPPRNQFGPAASNFPFGMVPPGMAIPMPSLGSLASAKMAGHP
STMQGYQGLMMPEGEMKMEPISQLGFPVVNQSSSSFQQMMNRPPFGPQM*
>OoW94
MEEEVEAANRAAVESCHRVLALLSQQQDPALLRSIASETGEACAKFRKVVSLLGNDGGAGGGRGGGGGHARG
RMAGRSRPAAVLREKGFLESSSGAGGGGGQLGMMMSGAATPSTSSAAHLRNRIGGSAPPDSLRGLDLVSSSS
KGGAHQFDPPKLVQPLSVQFQFGATAHRYPFQQHQHQQKLQAEMFKRSNSGISLKFDSPSATGTMSSAFMSS
LSMDGSVASLEGKPPFHLIGGPVASDPVNAHHAPKRRCTGRGEDGSGKCATTGRCHCSKRRKLRIKRSIKVPAIS
NKIADIPPDEYSWRKYGQKPIKGSPHPRGYYKCSSVRGCPARKHVERCVDDPAMLIVTYEGEHNHTRLPTQSAQ
T*
>OoW95
MLELGGDTNKLTNLVGGKRKGDKHSMDNHNLEEEAKESGNKRRKNAEHTGSTVAQAPHNDGHQWRKYGQ
KWISRAKHFRSYYRCANSKVQGCPATKTVQQMDSSGNGTSKLFNVDYYSQHTCRGDGIANPYVVDTAHHSTE
PINQTKCNSPTLEHEAHEVQDERFENLCMVQNMPEYLIEFELERAFEFTVNSPLGSEHWMFDDSIRCEQSPICI
WG*
>OoW96
MSARPPPPPRPRLALPPRSAAESLFTGAGDASPGPLTLASALFPSDPDGGVMTSSSAAAGATSFTQLLIGNLAAP
PPPLPPPQQQQQREAARGGGVARAGPALSVAPPAAAGSVFTVPPGLSPSGLLDSPGMLFSPAMGGFGMSHR
EALAQVTAQASHSPLRMFDHTEQPSFSAAPTSSEAMQHMNAAVNMTGISDMVTGPTNNENVAFQPAEASQ
RYQVNAPVDKPADDGYNWRKYGQKVVKGSDCPRSYYKCTHPNCPVKKKVEHAEDGQISEIIYKGKHNHQRPP
NKRAKDGSSSAADQNEQSNETTSGLSGIKRDQEAIYGMSEQLSGLSDGDDMDDGESRPHEADDKESDSKKRN
IQISSQRTSAEAKIIVQTTSEVDLLDDGYRWRKYGQKVVKGNPHPRSYYKCTYAGCNVRKHIERASSDPKAVITTY
EGKHNHEPPVGRGNNQNAGNAAPSSSSQQNMQNLSSNQASLTMADFNNINQRPIGVLQFKSEE*
>OoW97
MALDSVVPSYPSDLGSSRPTSTRTPQQQRVSPRKEERTWTTDTYAPYDDGHQWRKYGEKKLSNSNFPRFYYRC
TYKNDMKCPATKQVQQKDTNDPPLFSVTYFNHHTCNSSSKIVGSTPDSTGQSSSRKAISICFNSHGTTGEQPTFL
SSSASLLSPSIQSYRSNQQPDMNTYSRQFQWTDTSSSTSNAPVKTEADDYAEASASPNTTGALSRTLLPIGQSRC
IEYFHFL*

>OoW102
MFPSPGRAVMALGHHGAARQQATAMAAAASSTTTSAAAATVAFSFHQPSPTPPPHHHGILGYSPLVLDHHPT
TTTSSHAPSPPTLHHHALHAAAPPAAHPPRSSPPHPWSCEEGDRGRQMGKGAAATMGMGINDDGNNAAA
VSAAAAQHHLGVGAVRMKKAAGGGKTRRKVREPRFCFKTMSDVDVLDDGYKWRKYGQKVVKNTQHPRSYY
RCTQDNCRVKKRVERLAEDPRMVITTYEGRHVHSPSRDDDDAARASTEMTFIW*
>OoW104
MKILESFGHSDCQVVINMIEHQKALMVELRGMVIPILPSDNEQAKLALQLLGNILSCSDRAISMLELGGDTNKLT
NLVGGKRKGDNHSMDNHNLEEEAKESGNKRRKNAEHTGSTVAQAPHNDGHQWRKYGQKWISRAKHFRSYY
RCANSKVQGCPATKTVQQMDSSGNGTSKLFNVDYYSQHTCRGDGIANPYVVDTAHHSMEPINQTKCNSPTLE
HEAHEVQDERFENLCMVQNMPEYLIEFELERAFEFTVNSPLGSEHWMFDDSVRCEQSPICIWG*
>OoW107
MDIVVESPPVRDEKKVDVATIGGAPPIVFESFASSTKRDSTIIKKEEKMEAAKAEMGEVREENERLKTMLSRIVSD
YQSLHMHFLDVVKVKEQAAADDKAPPAAELAAVAADDDDEPDDLVSLSLCTRPNAAAAAAARRKGHERTSSS
SSSGGGHDDGRLSLGLSRARGVASDDDDKASRALPPPTTPVLNLSSDSSGDATAAAAEPNQPNKASRSSSDGA
GDGADDEVLQQQQAKKARVSVRVKCDTPTMNDGCQWRKYGQKISKGNPCPRAYYRCTVAPNCPVRKQVQR
CADDMSILITTYEGTHSHPLPPAAAAMASTTSAAAAMLTSGSTTSTMHGGGAHHHLPFTSAGGGGVGLLGPT
TISTATSCPTVTLDLTAPHSLLHPSSSSPYATAGYESSRALPAAWSSGYLAYGGAAAQPYYPAAKGIAPSPFGHHFG
LMGMATAAARPAPEQLFGGQTTSPYLQRAIGGGVAPAAVTDTIAKAITSDPSFQSVLAAAITSYMGRGGGAAA
QNK*
>OoW108
MQAQSRLAAASGGSGSGSSDIISFGGAGEEHEAVVRELTRGHELTARLRAEALRALRGQGQAEATATFILGEVSR
AFTVCLSIMASTSPSSPRPETPPDSAVSVGAAPLLPTRAAREDNVPRKRLLTASPYDDGYQWRKYGQKKINNTN
FPRSYYRCSYHRERRCPAQKHVQQRDGDDFPALHVVVYTHEHTCLQGAPVELPDATNSGAGVAAAASPDYFP
AGETPSSLRRRGAQVVGGRPQFVDDRAAMEERERQVLVSSLARVLQGRQCYDDPGVDSRGAVGAVSAVHAP
VAASSSSELPGPVDAAGEGLDVMDYDVTDALFWGPFGTDSNSYGKPDVDTLF*
>OoW109
MTKMSSMKADGSVPKRRRQDVQKVVVSLKDHKVEQGPPADSWSWRKYGQKPIKGSPHPRYHHLALIPHTYT
ALNVLVILQSFACALVLYITDNFTRIRLSGQLLVRFFLLYQSILLALFVDHAASRGYYKCSSYRGCPARKQVDKCRND
ASLLIITYTSDHNHDNYATTTANSVQEQAHNPDRSDAGDALSNGMSLAEVVTVASSKLSSEEESCDFFDELEELP
ASASPLPSLSFMVQESSFSDARTLL*
>OoW111
MNPLLLLLMNLTHASSLSRFRCRRISNKRVVNKLIMEYSNDWDLQALVRSCGTAVADSEPEPPAPSTRRAEAET
VVVGRAGGAPEFVGQPVRSAAAAAASFYDLEYLDVYHELPRAPFLVTAPSTSRERGEGEHEVLISFPAASTSGQ
GRKQPGRKPGVRTARPKRSKKSQLKKVVCEVPVADGGVSTDLWAWRKYGQKPIKGSPYPRGYYKCSSLKACM
ARKMVERSPEKPGVLVVTYIAEHCHAVPTQLNSLAGTTRNNKPASPDHHQQQPSPGGASTDEAATAAKPEDS
ADTCSMADDENDLWAPVEMDMDDFFGPFDDDLDHFLDDDGVLGRRLSL*
>OoW113
MDGGDIHLLLSILADGEEQARQLGEPAAAADDEYHGGGHGRGEEYYRGVARQLQCTFARAMAVARAIEAAA
GAGGGASGSRGTTGDRSDSPRSADESSGRTARDAVAQQERHHDTIKRRKGLPRWTENIRVPDASLEATPDDGF
SWRKYGQKDILGAKFPRGYYRCTYRNAQGCPATKQVQRSDADLAVFDVTYQGAHTCHQRQRRVPASPPAAG
DQPPPQADPSVELLVNFKHGLKVETNGLAPPPPPPPTNFHDGHDQHFCFPSMPPFHAGVGPPPPADDALGG
GCNFSSPFVPPAGSAAGSSYFSMEHSYEPRGQFVMSRGDSSELHEVVSAAAPSAMVDPAAAAAGGGEFDYPL
YHGEVDPHLPFPPLFGHASMYGQYRDA*
>OoW114

MAVTDSVCLSDEQEAVAVTEVAQVYELIKTQQPLLLVHQQPQQLAHGLLSHALRALNVALSVMNQPHHQHAS
SSAAAAAVPVMSMIKAEAATPANSSSPAADVADNHVVGKPRRSSPAKRRRINCEDKSSWVNHTVVPHEDGYQ
WRKYGEKNIQGTHFTRSYFRCTYRDDMGCQATKQVQQKDNNDPPNFQVTYSNDHTCNFGRTTTRIINNTNN
NPAAALHSLTANPNDPDDDDDDDTIFTKMIKQEKPAAWLPPPDLTAISNNSDETPVLHVCQEVAPCSSNSSVIS
HYADEFDQHQMLETTVMEEALGLGADLDDPYFYDPSLLVLYENLMNCY*
>OoW115
MASGGGGTAAAATTEKLIRRAQKSTNQLKALLAGGGGGRSSGAVEVILSDISDSLSQALASLVLRAASDDRLLPA
AAPPEASLLPSYGQSVVNSGGRSVSKRKAQRRSKADGSSRRIILERGDPDDSYPWRKYGQKDILGARFARSYYR
CAQMLGCSARKHVQQSDDDPSRLEITYIGAHTCGGHRPSSPATNPVDGTRCDAATSHRLLPAAAAAAPSAMQ
KWDEHVVASSDDMMVVCTPSMAASSWLFIPSPACSQSELLSEAEVPELRVRQEQDASPDDLVEVEEHKKPCD
ADDESLPLHDSVVPDFL*
>OoW116
MASPRLKREQSFDFEEASAQEAVGSASASYSPPGAGVFGISPPESSPRDGRKRRKDRPSWVKHTFTPHFDGHL
WRKYGQKNIKDSAFPRLYYRCSYREDRQCLASKLVQQENDDDPPLYRVTYTYEHTCNTTPVPTPDVVAEQPPP
GAAGDAYLLRFGSSAGGGGGGIHADAQQQTERERQQQYQQHSARRPFMMLSFDSGSHLHEQPHAFPPDG
QLPTAASPSSFTAAEVLAPPPAPLPTTTMNDGGDLFSTWDAFRYGLDDDHGHLGNHGYLPDDCNGGDDNY*
>OoW118
MENQSGQPQYAMADQGFHPFSPFMLAPSTMQQHVGSSSSTAVIQMAALPSHTCYGNIDLADDGFHWRMC
GQNTIQGGLVFSYQCALPNCGVRKSITRSADGQTIETVCRGYHNHPRQSLRWLGDGSERLEPISQDIVLLEASD
AAGAAAGPSVTGTGNGHGQSSGSSDSYRDDDGDLGIDGDASAGDANAVKSKQVPAPAKGIIVQTASEVDILD
NSARHENSQPRKKVRSKSTVWEEFEVVLIDGKVQTAECKHCKKCLSAKTSGGTSHLLRHLKICPAQHGTSSVQK
KCSSLTDLPIAKSWKDDQESSLDKIIRSIVSNLCPFSAMYSASFAQFLAGRNPSLNMVQQATVEEKFLSVFHNKKL
KLKDKITTTPGGVFLSLGEWQRLYFQVRVACLTVHFIDEDWRINRKTIRCSLSVFGKSDILSLYPHWQSDIFHAEK
VLKEVVRDWGLLDKLLGVTLQRSVDKKAPLHLEDDITGRNYLLSKCKLLSIPCMVDALHELMDSTVLDMESTWS
HYMTSSPERKQKYQEVLSQLHLDRPSLGSKGWYLTFYFSEAALQFIKSFPLPDVKPNYQSDPCEPSFDDLEATEN
YCKIARAAYHVIKVVSGSHNMTSDSYFHVIWSLRAAIQELPSMKNIGKVFDVAYMQKKFEKNWKKWYLWLSIA
VVLDPRYKLEFIERCFRQAFSHVAGMYFSEVRAKLYELYIQYSYVNEQSKEILDHNNNCSDIQISAPLHNKGQNST
TAQAAVEEFKELYEYLGGGQCPQDDSFDILKWWRGNSSAYPTLARMARDILAIPGCAVSAESAFDQCDQRAEL
FNGKLRPETTEALICAQSWIKSSGTADADDGNKNILF*
>OoW119
MGDVLRAQATAAADEVCVWPGELDEHLIGELLVDDGLFVPAAEHPDLYYSFDAGSSAAAAAAAPCNGGGSAE
HEPPRPAPAVSRALCSVYSGPTIRDIEKALLSSSSRSPYPSGHRYSSLYFRRVEAESKYTSKVRSCGGKMPADGYK
WRKYGQKSIKNNRHPRSYYKCTSSRCSAKKHVEKSTDDPEMLIVTYEGSHHHGPQPLFPPHIAQPPPTTSVVDF
SAAGAEPPSSSAAARKRKNDVRPGFSPTTSEDDVDGAGLRPGWPQDDETSRDDAELQRRGAAPRRVATDHS
CDDGGGSTSASSVARADAVTALSSDSPPTIWSCLDWPWSQETLFL*
>OoW121
MEGMEEANREAVQSCHRVLTLLSNPHSQLVPNKDLAAATGEAVAKFGSVASRLNNGNGLQGHARVRKIKKPL
PIFDSNLFLESPAVAAATAAKTSNPSPITSLQLFPRYHQMEGSSSKDPVRIPAQFPKRLLLDNPVVDSDGPSRGLPL
QLIQPVSVAPPAGTPHPALPSAHLHFIQQHQSYQRFQLMQQMKMQSEMIKRSGLGEQGGSNGGGKGVNLKF
DSSNCTASSSRSFLSSLSMEGSIASLDGSRSSRPFQLVSGSQTSSTPELGLMQRRRCTGREDGSGRCTTGSRCHC
AKKRKLRIRRSIKVPAISNKVADIPADEFSWRKYGQKPIKGSPHPRGYYKCSSVRGCPARKHVERCVDDPSMLIVT
YEGDHNHNRVLAAQPA*
>OoW125

MEGAMFNLPGRLDGLLLRHGSMLPKGAEEEIPLIKQDLEEIISVLHGHCSEPKLEDHAMVVRFWMKEVRELSY
DIEDCMDQYEHAATATQSRTGPNIRRRKFNRRRGNKIPWVPQKLMQRLWMANKIREFSLRAQEALQRHTMY
NNFGGITTASTTRGDVCFTTPWHPMQFREHTDNVHSVGIDADGMEAALNDLNKLKDLLAGIPTASLVQFREH
ANRVRGIHIDMEAILNKLENIPPGITTTTTARGDVSSTSSRQPTRFMESTCHVGIDAAVNKLENLLDVCGEEKLK
VVSIVGVGGLGKTTLAKKLYHKLRRQFECRAFVRTSQKTDMRRLLINILSQVLPHQSPDNWKAHSLISSIRTYLQ
DKRYLIIIDDLWATSTWDISKCSLPDGNNSSRILTTTEIDDLALQSCSYDLKFIFKMKPYCEDDSRKLFFSTIFGSHSK
CPPQVSETLYDIVRRCGGLPLAIVTVASLVASHLDKQEQWDYINKSLGYSLMANPTLEGMKQLLNLCYNNLPQH
LKACMLYLSMYQEDHIIWKDDLVSQWIAEGFICAIEGHDKEEISRAYFDELVDRKIIQPVHIDDNGEVLSCVVHH
MVLNFVTYKSIEENFIIAIDHSQATTRFSDKVRRLSIHFGNVEDATPPTNMRLSQVRTVAFFGILKNMPFIMEFRL
IKVLVLHFLDDGDSIGIFDLTKISELVQLRYLKVTSNVILKLPTQIQGLQYLVTLKIDGKISAIPSDIIHLPGLLHLTLPAK
TNLPNGIVHMTSLRTIGYFDLSCNSEENLWSLGELTNLRDLQLTYSEIHSDNLKNNMKYLGSILGKLRNLKSIILSP
ASSSYADTLHIDRATSTRISVNGWNSMSSPPALLQRFELLPCVCIFSNLPNWIGQLGNLCILKVGIGEITSNVVNVL
GGLLALTVLSLYVHTKPAERIVFDNVGFSILKYFKFICSVPWMKFEAGAMPNLRKLKLGFDVHRADQHDIIPVGIE
HLSGLEEISAKIRVACTAHDHCRRFAESALTNAFRMHPGRPSVNIRCVDWTFDGKDDSNAGTREEECRTLQKQ
QNIVKESSTEKCAVLEKDPGRGANKSVESREKPHMVGRHLKSNTEDDGLSWIKYDQKDILGAKFPRAYFRCTH
WSSTKKKGCMATKEVQRDDDDPLLFDIVYHGDHTCAQTNNEQPVRSLTRSTDEQSQQSGLELSSPYDWHGH
KLSAKAQVTKMTTQVQVRLSSVQDVEPLWDGHGWRKYGQKDIVGAKFPRFYFRCTHRNtqGCVATKQMQRR
DADPLLFDVTYHGKHTC
